# Generation of hypoimmunogenic cardiomyocytes from human induced pluripotent stem cells with TAPBP knockout

**DOI:** 10.1016/j.isci.2026.117933

**Published:** 2026-10-17

**Authors:** Yuki Yamamoto, Rei Ohno, Kayoko Shoda, Toshihiro Suzuki, Kuniko Momoi, Shohei Fujii, Yuka Kenmochi, Toshiaki Yoshikawa, Manami Shimomura, Nobuo Tsukamoto, Kazunobu Ohnuki, Hidenori Tani, Yusuke Soma, Keiichi Fukuda, Tetsuya Nakatsura, Shugo Tohyama

**Affiliations:** 1 Department of Clinical Regenerative Medicine, Fujita Medical Innovation Center, Fujita Health University, Ota-ku, Tokyo 144-0041, Japan; 2 Heartseed Inc., Minato-ku, Tokyo 105-0023, Japan; 3 Division of Cancer Immunotherapy, Exploratory Oncology Research and Clinical Trial Center, National Cancer Center, Kashiwa, Chiba 277-8577, Japan; 4 Kanagawa Institute of Industrial Science and Technology (KISTEC), Kawasaki, Kanagawa 210-0821, Japan; 5 Keio University Regenerative Medicine Research Center, Kawasaki, Kanagawa 210-0821, Japan; 6 Department of Cardiology, Keio University School of Medicine, Shinjuku-ku, Tokyo 160-8582, Japan

**Keywords:** cardiomyocytes, hiPSC-CMs, TAP-binding protein, TAPBP, hypoimmunogenic cells, universal cells

## Abstract

Immunological rejection is a challenge for the transplantation of cardiomyocytes (CMs) derived from human induced pluripotent stem cells (hiPSCs). TAP-binding protein knockout (TAPBP-KO) hiPSC-derived CMs (hiPSC-CMs) were used to reduce human leukocyte antigen class I expression and their hypoimmunogenic potential was determined. TAPBP-KO hiPSC-CMs exhibited CM differentiation efficiency and functional properties comparable to those of controls. The immunological responses against cytotoxic T lymphocytes (CTLs) or natural killer cells (NK cells) were measured using the production of inflammatory cytokines, the expression of degranulation markers, and cell viability upon co-cultivation with hiPSC-CMs. The CTL-mediated cytotoxicity was lower in TAPBP-KO hiPSC-CMs than in controls. TAPBP-KO hiPSC-CMs did not induce cytotoxicity from NK cells. This approach can preclude immunological rejection from CTLs without initiating the innate immune response from NK cells, thereby expanding the access to cell therapy for patients with heart failure.

## Highlights

• CRISPR-Cas9-mediated TAPBP knockout reduces HLA class I expression in hiPSC-CMs
• TAPBP-KO hiPSCs maintain stemness and cardiac differentiation potential
• TAPBP-KO hiPSC-CMs preserve structural and contractile functions
• TAPBP-KO hiPSC-CMs evade CTLs without triggering NK-cell cytotoxicity

## Graphical abstract

**Figure:**
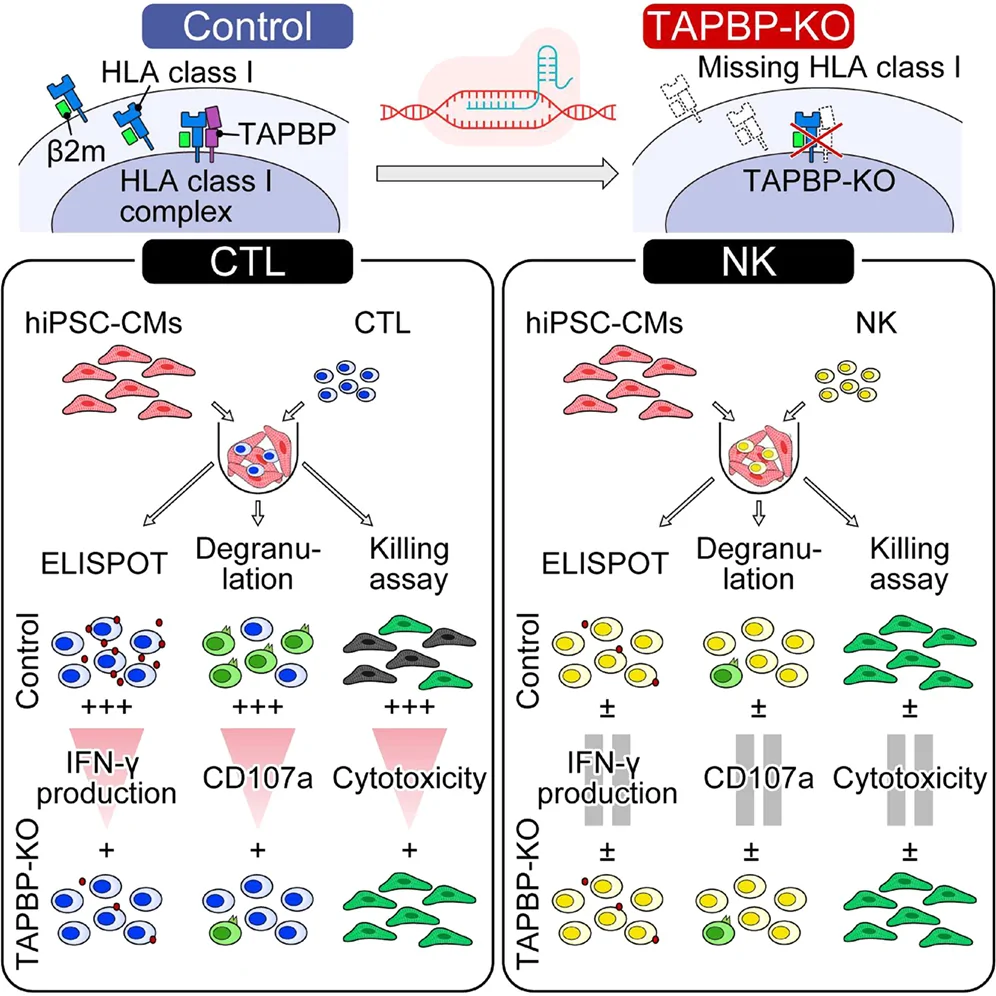

## Teaser

Molecular biology; Immunology; Cell biology; Stem cells research

## Introduction

Globally, heart failure is a common chronic disease, with the number of patients exceeding 64 million in 2017, which was nearly double that of 1990; this number is expected to increase over the next few decades.^1^^,^^2^ Heart transplantation is the only radical therapy against severe heart failure that is intractable to medication and device therapies. Despite the sophistication of the allocation system, the number of donors is still limited. In Japan, the number of annual heart transplantations is only approximately 100, and the average waiting time for heart transplantations is over five years due to the serious donor shortage.^3^^,^^4^

To overcome this limitation, transplantation of cardiomyocytes (CMs) derived from human induced pluripotent stem cells (hiPSCs) is expected to be a promising solution.^5^^,^^6^ Although autologous hiPSC-CMs have the potential to mitigate the risk of immunological rejection, there are various challenges associated with delivering the cell therapy to the patients, including the long manufacturing lead time, and ensuring the safety and quality control of the reproducible procedures under the good manufacturing practice (GMP) requirements.^7^^,^^8^ The prospect of allogeneic transplantation offers a potential solution to these challenges through large-scale manufacturing and off-the-shelf delivery of hiPSC-CMs from validated donor cells. Recently, preclinical studies with non-human primates (NHPs) and clinical studies have been carried out by several groups resulting in positive outcomes.^6^^,^^9^^,^^10^^,^^11^ However, the risk of immunological rejection remains. A strategy of human leukocyte antigens (HLA) matching was implemented to reduce immunological rejection; however, this strategy was restricted to specific populations due to the wide variety of HLA.^12^ Consequently, the patients are forced to take immunosuppressants, balancing the risk of graft loss against the risk of infection.^13^^,^^14^^,^^15^ Given these issues, hiPSCs with low immunogenicity can potentially prevent graft loss due to donor-host mismatching and enhance treatment efficiency.

The immunological rejections upon transplantation are facilitated by the expression of highly polymorphic HLA.^16^^,^^17^ Recognition of mismatched HLA class I molecules can initiate cell killing by the cytotoxic T cells (cytotoxic T lymphocytes, CTLs; CD8^+^ T cells), which is the most relevant and rapid cellular event for allograft immune rejection. Conversely, cells that lack HLA class I expression can evade CTL-mediated cytotoxicity, even in the allograft transplantation settings. Thus, various groups have attempted to establish HLA class I-null hiPSCs by the disruption of each individual HLA class I genes or β2-microglobulin, an indispensable protein in maintaining HLA class I expression.^13^^,^^18^ Although diminishing the HLA class I reduced the cytotoxicity from CTLs as hypothesized, it encouraged cytotoxicity by natural killer cells (NK cells).^18^^,^^19^^,^^20^^,^^21^ This is considered to be due to missing-self signaling as HLA class I is one of the inhibitory ligands for NK cells.

Tapasin is a 48-kDa transmembrane glycoprotein encoded by the TAP-binding protein (*TAPBP*) gene that acts as a chaperone in antigen presentation machinery by HLA class I molecules.^22^^,^^23^ Tapasin bridges the HLA class I molecule and the TAP transporter, which stabilizes unstable HLA class I with low-affinity peptides and catalyzes peptide exchange for high-affinity HLA class I peptides. The Tapasin-deficient mice showed lower expression of HLA class I, resulting in immunodeficiency from CTLs.^24^ It was hypothesized that disruption of TAPBP in hiPSC-CMs would provide sufficiently low immunogenic potential by reducing their HLA class I expression without activating NK cells.

## Results

### *TAPBP*-deficient hiPSCs were generated using the CRISPR-Cas9 system

The *TAPBP*-knockout (KO) hiPSCs were generated using the CRISPR-Cas9 system, in which a plasmid, pX330-*TAPBP*, encoding both *TAPBP* gRNA and the *hCas9* gene, was transduced into FfI14 cells by electroporation (Figures 1A and S1A). A random selection of 56 colonies showed various levels of HLA class I expression, and one clone with the lowest expression of HLA class I was selected for further analysis (Figures 1B and S1B). Then, the loss of TAPBP protein expression was confirmed using western blotting (Figures 1C and S1C). Based on the Sanger sequence analysis, a single nucleotide was inserted in the target sequence. TAPBP-KO hiPSCs formed a colony-like morphology and expressed pluripotent markers (Figures 1D and S2A). No abnormality was identified in the karyotype (Figure 1E). The cell expansion rate was equivalent between control and TAPBP-KO hiPSCs (Figure S2B).

**Figure 1:**
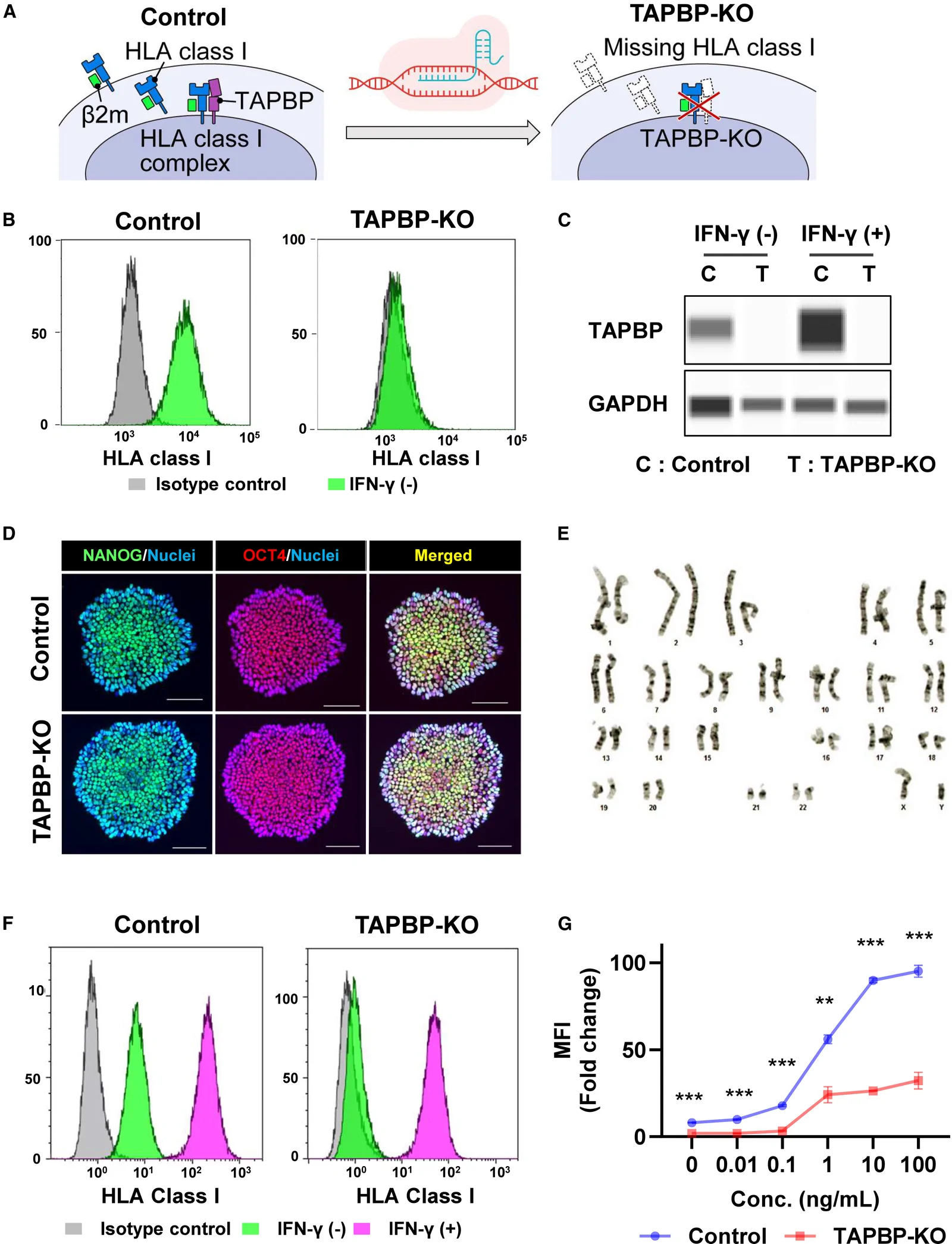
TAPBP-KO hiPSCs established using the CRISPR-Cas9 system (A) Schematic representation of the *TAPBP* gene knockout using CRISPR-Cas9 to disrupt the expression of HLA class I. (B) The representative histogram using flow cytometry shows the minimum HLA class I expression in the selected clone of TAPBP-KO hiPSCs. (C) Loss of TAPBP expression in TAPBP-KO hiPSCs was evaluated by western blotting. GAPDH is recruited as an internal control. (D) The representative immunostaining shows the remaining pluripotent markers in TAPBP-KO hiPSCs. Control and TAPBP-KO hiPSCs are stained with NANOG (green), OCT4 (red), and Hoechst33432 (blue); scale bars, 100 μm. (E) Representative results of karyotype. (F) The representative histograms using flow cytometry indicate enhanced HLA class I expression after treatment of 10 ng/mL IFN-γ (pink) compared with non-treatment (green) in control and TAPBP-KO hiPSCs. (G) Dose-dependent responses against IFN-γ stimulation were evaluated by flow cytometry (*n* = 3). Each plot shows the relative mean fluorescence intensity (MFI) of HLA class I against isotype control. The data are presented as mean ± SEM; ∗∗∗*p* < 0.001, ∗∗*p* < 0.01 using an unpaired *t* test.

### TAPBP-KO hiPSCs responded to IFN-γ stimulation in a dose-dependent manner

Interferon gamma (IFN-γ) has been reported to be able to up-regulate HLA class I expression.^25^ Control or TAPBP-KO hiPSCs were treated with IFN-γ for 48 h, and the expression levels of HLA class I in both control and TAPBP-KO hiPSCs were measured using flow cytometry. The expression level of HLA class I in TAPBP-KO hiPSCs was weaker than that in controls (Figure 1F). A dose escalation study of IFN-γ also confirmed that TAPBP-KO hiPSCs had less HLA class I expression in each concentration and reached a plateau at a lower dose than controls (Figure 1G). In contrast to HLA class I expression, HLA class II expression level remained low, even in the presence of IFN-γ (Figures S2C and S2D).

### TAPBP-KO hiPSCs were differentiated into CMs

Both control and TAPBP-KO hiPSCs were differentiated into CMs using a previously reported method.^26^^,^^27^^,^^28^^,^^29^ hiPSC-CMs were metabolically selected by glucose- and glutamine-depleted, lactate-supplemented media.^27^^,^^28^^,^^29^ Although the percentages of cardiac troponin T (cTnT)-positive cells were varied at day 10, they reached over 95% on day 17 after the metabolic selection in both control and TAPBP-KO hiPSC-CMs (Figure 2A).

**Figure 2:**
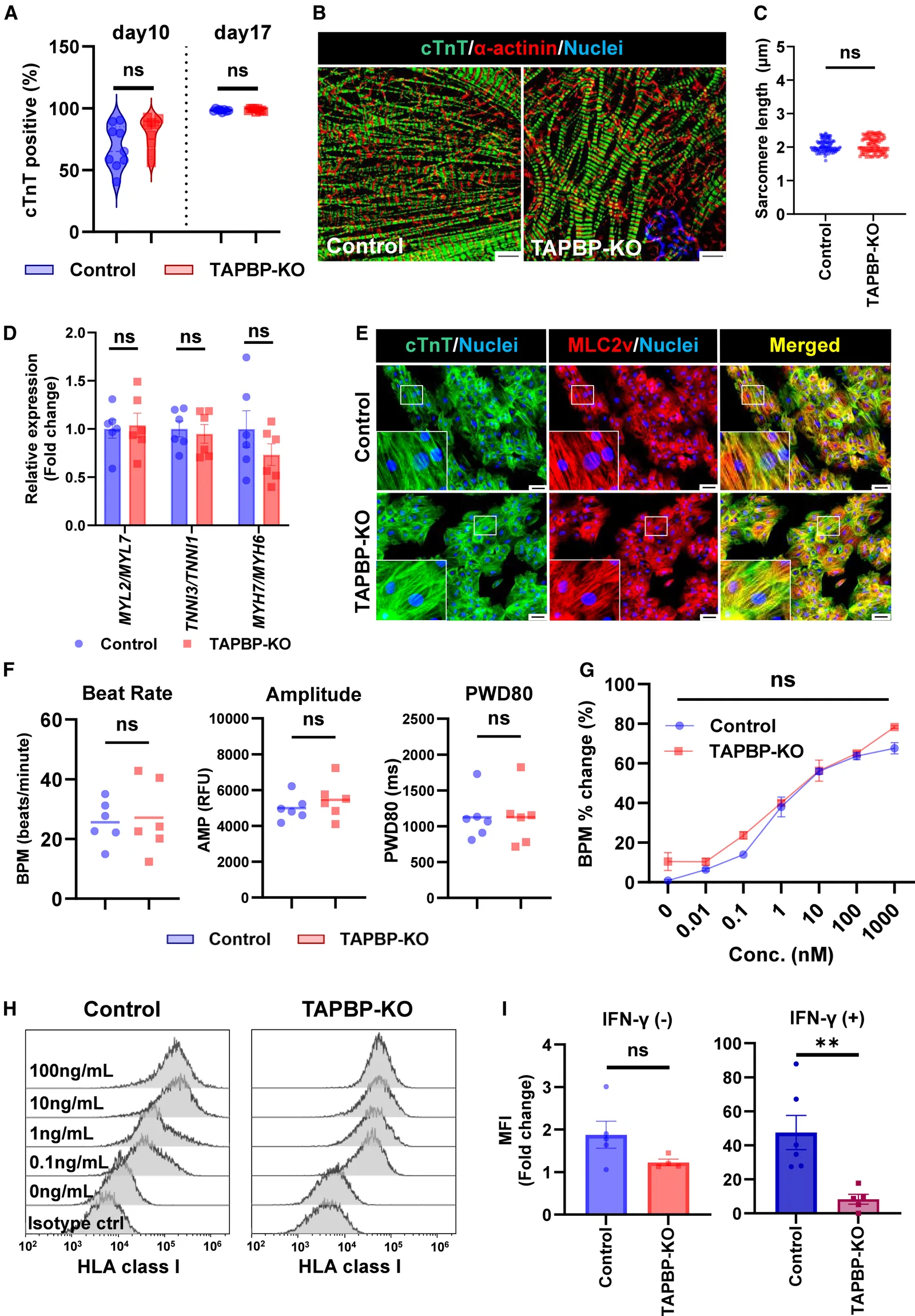
Differentiation of hiPSC-CMs from TAPBP-KO hiPSCs (A) The plots show the variety of cTnT-positive population on days 10 and 17. Control (*n* = 8) and TAPBP-KO (*n* = 8) hiPSC-CMs on day 10, and control (*n* = 7) and TAPBP-KO (*n* = 8) hiPSC-CMs on day 17, from independent experiments; ns *p* > 0.05 using unpaired *t* tests. (B) Immunological staining with cTnT (green) and α-actinin (red), and Hoechst 33432 (blue); scale bars, 5 μm. (C) Quantification of sarcomere length (*n* > 100); ns *p* > 0.05 using unpaired *t* test. (D) Expression ratio of *MYL2/MYL7*, *TNNI3/TNNI1*, and *MYH7/MYH6* was evaluated by RT-qPCR (*n* = 6, independent experiments). The expression is normalized to *GAPDH*. The plots indicate data points, and the bars are presented as mean ± SEM; ns *p* > 0.05 using unpaired *t* tests. (E) Immunological staining with cTnT (green), MLC2v (red), and Hoechst 33432 (blue); scale bars, 100 μm. (F) Beating characteristics were evaluated by Ca^2+^ fluctuation through FDSS (*n* = 6, independent experiments). BPM: beats per minute, AMP: peak amplitude; PWD80: peak width duration; ns *p* > 0.05 using unpaired *t* tests. (G) Increase in beating rate in a dose-dependent manner to the β-adrenergic receptor agonist (isoproterenol) was evaluated by FDSS (*n* = 3). The data are presented as mean ± SEM; ns *p* > 0.05 using unpaired *t* tests at each concentration. (H) Representative histograms using flow cytometry show dose-dependent elevation of HLA class I against IFN-γ stimulation. (I) HLA class I expression with or without 10 ng/mL of IFN-γ stimulation was evaluated by flow cytometry. The plots show the differences of mean fluorescent intensity (MFI) against control hiPSC-CMs without IFN-γ treatment. In IFN-γ (−), control (*n* = 5) and TAPBP-KO (*n* = 4) hiPSC-CMs and in IFN-γ (+) control (*n* = 6) and TAPBP-KO (*n* = 5) hiPSC-CMs. The plots show values from independent experiments and the bars are presented as mean ± SEM; ns *p* > 0.05, ∗∗*p* < 0.01 using unpaired *t* tests.

Immunostaining for cTnT and α-actinin demonstrated comparable sarcomere organization and alignment between control and TAPBP-KO hiPSC-CMs (Figures 2B and 2C). In addition, quantitative reverse-transcription PCR (RT-qPCR) analysis showed similar expressions of sarcomeric protein isoforms, including *MYL2/MYL7*, *TNNI3/TNNI1*, and *MYH7/MYH6*, in the two groups (Figure 2D). MLC2v expression was also comparable, indicating successful differentiation into ventricular CMs (Figure 2E). Furthermore, MitoTracker staining revealed similar mitochondrial distribution patterns between control and TAPBP-KO hiPSC-CMs (Figure S3A). Collectively, these findings indicate that TAPBP disruption did not produce detectable differences in the structural characteristics evaluated in this study.

The contraction characteristics were also evaluated from Ca^2+^ transients using the Functional Drug Screening System (FDSS); there were no differences in beat rate, amplitude, and peak width duration at 80% repolarization (PWD80) (Figure 2F). The morphology and the contraction of control and TAPBP-KO hiPSC-CMs were recorded (Videos S1 and S2). Contractile properties were further measured using the SI8000 live-cell motion system (Figure S3B). When the adrenergic response was measured by adding isoproterenol, the beat rate was increased in a dose-dependent manner in both control and TAPBP-KO hiPSC-CMs (Figure 2G). This demonstrated that TAPBP-KO hiPSC-CMs exhibited similar contractile characteristics to controls.

Video S1. Representative video showing the spontaneous contraction of control hiPSC-CMs

Video S2. Representative video showing the spontaneous contraction of TAPBP-KO hiPSC-CMs

Taken together, the disruption of *TAPBP* did not affect the differentiation efficiency and the characteristics of hiPSC-CMs.

### Lower expression of HLA class I in TAPBP-KO hiPSC-CMs

The flow cytometry analysis indicated that control hiPSC-CMs expressed a low level of HLA class I, with even less on TAPBP-KO hiPSC-CMs. Under the treatment with IFN-γ, the HLA class I expression was enhanced in a dose-dependent manner (Figure 2H). The expression level of HLA class I in TAPBP-KO hiPSC-CMs was substantially lower than in controls, with or without IFN-γ (Figure 2I). In addition, the expression of several immunogenicity-associated molecules, including HLA-C, HLA-E, HLA-F, HLA class II, PD-L1, and PD-L2, with or without IFN-γ stimulation, was comparable between control and TAPBP-KO hiPSC-CMs (Figure S4).

### TAPBP-KO attenuated the cytotoxicity caused by allogeneic CTLs

The immunological rejection of donor CMs by host CTLs depends on HLA mismatch. Allogeneic CTLs were isolated from PBMCs of three donors whose HLA haplotypes were mismatched with hiPSC-CMs (Table 1). The immune responses of allogeneic CTLs were evaluated using an enzyme-linked immunospot (ELISpot) assay for IFN-γ and a degranulation assay with cell-surface CD107a staining. The degranulation assay detected the transient surface expression of CD107a on the CTLs caused by the cell degranulation upon antigenic stimulation, which was followed by perforin and granzyme exocytosis. The cytotoxicity of CTLs against hiPSC-CMs was evaluated using a calcein-based cell killing assay after the co-cultivation of allogeneic CTLs and hiPSC-CMs (Figure 3A).

**Table 1:** HLA Haplotypes of the cells used in this study

Cell type	Cell name	HLA-A	HLA-B	HLA-C
hiPSC-CMs	control TAPBP-KO (FfI14)	24:02	52:01	12:02
CTLs	CTL A	03:01/68:01	14:02/35:43	01:02/15:05
	CTL B	30:02/33:01	07:02/14:02	08:02/15:05
	CTL C	02:07/33:03	44:03/46:01	01:02/14:03
NK cells	NK D	03:01/68:01	14:02/35:43	01:02/15:05
	NK E	30:02/33:01	07:02/14:02	08:02/15:05
	NK F	01:01/02:01	14:02/57:01	07:01/08:02

**Figure 3:**
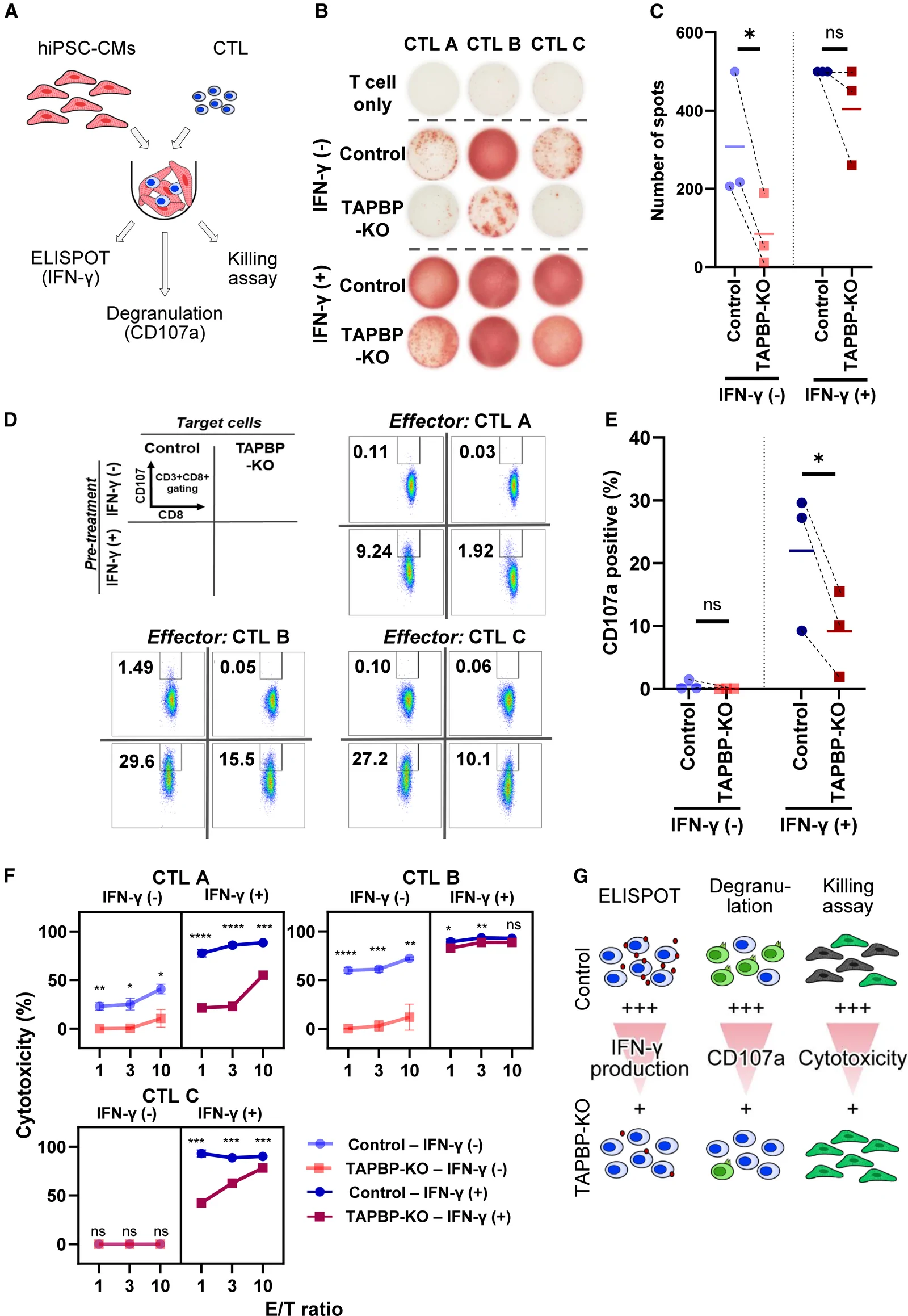
The cytotoxicity of CTLs against hiPSC-CMs after the co-cultivation of allogeneic CTLs and hiPSC-CMs (A) Schematic of the analysis items in the immunological responses of CTLs co-cultivated with control or TAPBP-KO hiPSC-CMs. (B) Representative well images for the IFN-γ co-culture of control or TAPBP-KO hiPSC-CMs with CTLs from three independent donors measured using the ELISpot assay. (C) The number of IFN-γ-producing cells was calculated by the ELISpot assay. Plots and solid lines show individual donor data and the mean of them, respectively. Dashed lines connect data points derived from the same donor. The *p* values are evaluated using a paired *t* test, with *n* = 3 donors; ∗*p* < 0.05, ns *p* > 0.05. (D) The plots of flow cytometry against CD107a after co-cultivation of control or TAPBP-KO hiPSC-CMs with CTLs from three independent donors. (E) The change in the CD107a-positive population was evaluated by flow cytometry. Plots and solid lines show individual donor data and mean, respectively. Dashed lines indicate data points derived from the same donor. The *p* values are obtained using a paired *t* test, with *n* = 3 donors; ∗*p* < 0.05, ns *p* > 0.05. (F) The change in luminescence using calcein-AM after the incubation of control and TAPBP-KO hiPSC-CMs with CTLs. The relative survival ratio in SP40-treated or mock was calculated. E/T ratio stands for effector (CTLs)/target (hiPSC-CMs) ratio. Data are represented as means ± SEMs (*n* = 3) and the *p* values are evaluated using unpaired *t* tests; ∗∗∗∗*p* < 0.0001, ∗∗∗*p* < 0.001, ∗∗*p* < 0.01, ∗*p* < 0.05, ns *p* > 0.05. (G) Schematic describing the result of CTL immunological responses after co-cultivation with control or TAPBP-KO hiPSC-CMs.

The production of IFN-γ was measured using an ELISpot assay, when allogeneic CTLs were co-cultured with hiPSC-CMs treated with or without IFN-γ (Figures 3B and 3C). Although the number and size of IFN-γ spots varied depending on the CTL donor, the number of spots was substantially lower in TAPBP-KO hiPSC-CMs than in controls. Whereas the IFN-γ production from CTLs increased to a saturation level against control hiPSC-CMs after IFN-γ pre-treatment in all three donors, the intensity of the spots was much lower in TAPBP-KO hiPSC-CMs.

The degranulation of CTLs co-cultured with hiPSC-CMs in the presence of an anti-CD107a antibody was measured (Figures 3D and 3E). Under co-cultivation with either control or TAPBP-KO hiPSC-CMs, CTLs showed low levels of CD107a positivity. However, pre-treatment of control hiPSC-CMs with IFN-γ markedly increased CD107a positivity. With IFN-γ pre-treatment, CTL degranulation against TAPBP-KO hiPSC-CMs was consistently lower than in controls: the percentage of CD107a-positive CTLs against control hiPSC-CMs vs. TAPBP-KO hiPSC-CMs was 9.24% vs. 1.92% in donor A, 29.6% vs. 15.5% in donor B, and 27.2% vs. 10.1% in donor C, respectively.

Cell-mediated cytotoxicity against hiPSC-CMs was measured using the viability of calcein-AM-labeled hiPSC-CMs after co-cultivation with CTLs (Figure 3F). Without IFN-γ pretreatment, a significant reduction in cytotoxicity was observed in TAPBP-KO hiPSC-CMs compared to controls for donors A and B. In contrast, donor C showed no statistical difference due to the absence of spontaneous cytotoxicity. Following the IFN-γ pretreatment, CTLs from donors A and C exhibited enhanced cytotoxicity, which remained significantly lower against TAPBP-KO hiPSC-CMs than against control hiPSC-CMs. For donor B, the cytotoxicity reached a plateau at an effector-to-target (E/T) ratio of 10 in both control and TAPBP-KO hiPSC-CMs.

To evaluate the effects of co-culture with CTLs on surviving hiPSC-CMs, we analyzed changes in the beating rate of hiPSC-CMs. Although co-cultivation with CTLs increased the beating rate of hiPSC-CMs, the increase was significantly attenuated in TAPBP-KO hiPSC-CMs compared with controls (Figure S5).

Despite donor diversity, these results demonstrated that TAPBP-KO hiPSC-CMs evaded immunological recognition by CTLs, thereby preventing IFN-γ production, cell degranulation, and cytotoxicity (Figure 3G).

### TAPBP-KO hiPSC-CMs did not initiate cytotoxicity from NK cells

NK cells are known as the innate counterparts of CTLs in cytotoxic cytokine production in an antigen-independent manner. NK cells can recognize altered cells, such as virus-infected cells and malignant cells, whose HLA class I expression is downregulated. Since TAPBP-KO hiPSC-CMs had lower expression of HLA class I (Figures 2H and 2I), there were potential concerns that TAPBP-KO hiPSC-CMs may trigger NK-mediated cytotoxicity. To assess NK-mediated cytotoxicity, CD56-positive NK cells were isolated and expanded from three independent donors, and co-cultured with hiPSC-CMs. Then, NK-mediated cytotoxicity was evaluated using ELISpot, degranulation, and killing assays (Figure 4A and Table 1). K562, a myelogenous leukemia cell line, was used as a positive control in the series of assays with NK cells.

**Figure 4:**
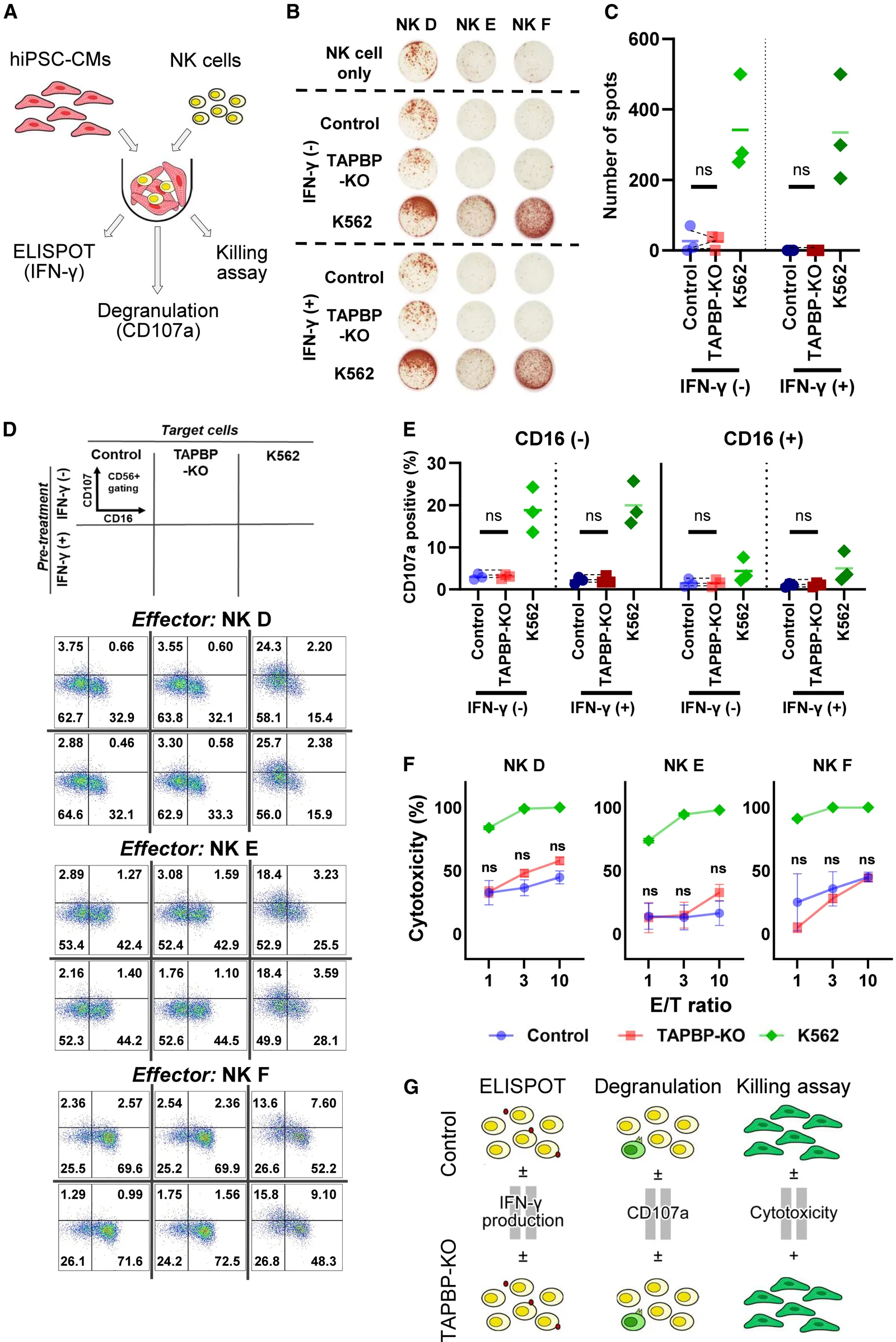
The cytotoxicity of NK cells against hiPSC-CMs after the co-cultivation of NK cells and hiPSC-CMs (A) Schematic of the analysis items in the immunological responses of NK cells co-cultivated with control or TAPBP-KO hiPSC-CMs. (B) Representative well images of IFN-γ produced by co-cultivation of control or TAPBP-KO hiPSC-CMs with NK cells from three independent donors measured using the ELISpot assay. (C) The number of IFN-γ-producing cells was calculated by the ELISpot assay. Plots show the number of spots for each donor, and lines connect data points derived from the same donor. K562 is used as a positive assay control. The *p* values are evaluated using a paired *t* test, with *n* = 3 donors; ns *p* > 0.05. (D) The plots of flow cytometry against CD107a after co-cultivation of control or TAPBP-KO hiPSC-CMs with NK cells from three independent donors. (E) The change in the CD107a-positive population in the CD16(+) or CD16(−) cells was evaluated by flow cytometry. Dashed lines indicate data points derived from the same donor. K562 is used as a positive assay control. The *p* values are evaluated using a paired *t* test, with *n* = 3 donors; ns *p* > 0.05. (F) The change in fluorescence using calcein-AM after the incubation of control and TAPBP-KO hiPSC-CMs with NK cells. The relative survival ratio of SP40-treated/mock is calculated. K562 is used as a positive assay control. E/T ratio stands for effector (NK cells)/target (hiPSC-CMs) ratio. Data are represented as means ± SEM (*n* = 3); ns *p* > 0.05 using unpaired *t* tests at each E/T ratio. (G) Schematic of the result of NK immunological responses after co-cultivation with control or TAPBP-KO hiPSC-CMs.

First, the production of IFN-γ from NK cells was measured using the ELISpot assay upon co-cultivation with control or TAPBP-KO hiPSC-CMs with or without IFN-γ pre-treatment (Figures 4B and 4C). The number of spots was equivalent between wells of NK cells only and in co-culture with control and TAPBP-KO hiPSC-CMs, whereas more IFN-γ spots were detected against K562. The results obtained from the experiments with and without IFN-γ treatment were comparable or sometimes indicated attenuation under IFN-γ pre-treatment conditions.

The degranulation assay with CD107a staining showed similar results (Figures 4D and 4E). After co-cultivation, NK cells were identified by gating the CD56-positive fraction, followed by a comprehensive analysis of the percentage of CD107a-positive cells in CD16-negative or -positive cells. Contrary to the results in the allogeneic CTLs, a marked degranulation of NK cells was not detected in control and TAPBP-KO hiPSC-CMs, regardless of their CD16 expression. K562 cells demonstrated elevated CD107a expression, particularly within the CD16-negative subset, in accordance with prior observations.^30^

The NK cell-mediated cytotoxicity was evaluated using the viability of calcein-AM positive hiPSC-CMs after co-cultivation with NK cells and target cells (Figure 4F). Although the percentage of cytotoxicity increased gradually due to the E/T ratio, the percentage of cytotoxicity was comparable between control and TAPBP-KO hiPSC-CMs. We also evaluated changes in the beating rate following co-culture with NK cells. No significant difference was observed between control and TAPBP-KO hiPSC-CMs (Figure S6A).

To comprehend the mechanism by which NK cells exhibited minimal cytotoxicity toward hiPSC-CMs despite reduced HLA class I expression, we evaluated the expression of activating ligands for NK cells. Unlike K562 cells, both control and TAPBP-KO hiPSC-CMs lacked the expression of representative activating ligands, including ULBP and MICA/MICB (Figure S6B).

These results demonstrate that the reduced expression of HLA class I in TAPBP-KO hiPSC-CMs did not initiate the innate immune response by NK cells (Figure 4G).

## Discussion

Recently, several research groups have attempted to reduce immunological rejection upon transplantation of hiPSC-related products. For example, a bank of hiPSCs with a homozygous HLA haplotype has been established to cover a certain population and has been released for use in a clinical setting.^31^ Because of the extremely wide variety of HLA worldwide, it would be applicable to a limited number of patients.^32^ Therefore, the interest of researchers has shifted to finding hypoimmunogenic hiPSCs that have an absence of HLA expression rather than matching HLA haplotype within the general population. Knocking down the expression of HLA class I is the most widely used strategy for this purpose. However, this approach triggers NK cell-mediated immunological rejection because HLA class I serves as one of the inhibitory ligands for NK cells.

To address these challenges in generating hypoimmunogenic cells, some research groups used a complicated gene-editing strategy that knocked down individual HLA and CIITA to keep the part of the HLA signaling that avoided detection by the NK cells.^33^^,^^34^ For example, Xu et al. reported that by knocking down HLA-A/B and CIITA while keeping HLA-C made it possible to escape from both CTLs and NK cells.^35^ Xiao et al. reduced the immunological response by knocking down HLAs and CIITA and overexpressing PD-L1, HLA-G, and CD47 to escape from CTLs, NK cells, and macrophages.^36^ However, the complicated gene-editing strategy increased off-targeting risks due to the multiple gene modifications, suggesting that a simple gene modification strategy was preferable for safety and quality control in a clinical setting.^37^ Moreover, we demonstrated that transplanted CMs derived from MHC class I-disrupted and CD47-overexpressed iPSCs did not engraft effectively in the heart of NHPs, suggesting that alternative hypoimmunogenic strategies are required.^15^ To date, only a limited number of studies have attempted to generate hypoimmunogenic cells by targeting the antigen-processing machinery responsible for HLA class I expression. Di et al. reported that disruption of TAPBP in human embryonic stem cells reduced immunological responses *in vivo* following intramuscular injection into BALB/c mice; however, their evaluation was restricted to xenotransplantation in immunocompetent mice.^38^

In the current study, we hypothesized that disruption of TAPBP reduced the expression of the HLA class I of hiPSC-CMs, resulting in suppressed immunological rejection by CTLs without the initiation of the innate immune responses by NK cells. TAPBP-KO hiPSCs were established using the CRISPR-Cas9 system. TAPBP-KO hiPSCs maintained stemness and differentiation potential into CMs, and TAPBP-KO hiPSC-CMs exhibited equivalent cardiac function to that of controls (Figure 2). While HLA class I expression remained stable at the hiPSC stage, it was profoundly diminished following differentiation into CMs, presenting an intriguing characteristic of these cells (Figures 1F and 2I). This reduction is considered to mimic the physiological regulation of HLA class I expression, where the heart exhibited lower HLA expression levels.^39^ Although the level of expression varied depending on TAPBP status, stimulation with IFN-γ induced an increase in HLA class I expression in both hiPSCs and hiPSC-CMs (Figures 1F and 2H). This may also mimic the inflammatory condition *in vivo.*^40^

The CTLs recognize HLA class I molecules to distinguish self or non-self. When CTLs bind to allogeneic HLA class I, alloreactive TCR signaling initiates the activation of CTLs, followed by cytotoxic activity. To evaluate the CTL-mediated immunological responses, hiPSC-CMs were co-cultured with CTLs generated from three donors with HLA haplotypes mismatched to those of hiPSC-CMs. Donor B-derived CTLs showed higher cytotoxicity in the ELISpot and killing assays compared to others, suggesting there was donor variety in CTLs. Despite the wide variety of donors observed, the correlation between CTL-mediated cytotoxicity and HLA class I expression levels remained consistent; TAPBP-KO hiPSC-CMs exhibited lower HLA class I expression and showed reduced cytotoxicity from CTLs compared to controls, regardless of IFN-γ stimulation (Figures 2I and 3).

As HLA class I is a suppressive ligand for NK cells that binds to killer Ig-like receptors (KIRs) and NKG2A, there is a concern that disruption of HLA class I expression could initiate the innate immune response from NK cells.^41^ The activation of NK cells *in vitro* was evaluated, revealing that NK cells exhibited only limited cytotoxicity toward both control and TAPBP-KO hiPSC-CMs compared with K562 cells. Because NK cell activation is determined by the balance between activating and inhibitory signals, we examined the expression of NK cell-activating ligands (Figure S6B). Representative activating ligands, including ULBP and MICA/MICB, were absent in both control and TAPBP-KO hiPSC-CMs, which likely explains their low susceptibility to NK cell-mediated cytotoxicity. Although the low baseline NK cell response to control hiPSC-CMs limits evaluation of the specific contribution of TAPBP deficiency, the preservation of minimal NK cell-mediated cytotoxicity despite markedly reduced HLA class I expression supports the potential utility of TAPBP-KO hiPSC-CMs for clinical transplantation.

hiPSCs with lower HLA class I were established by the disruption of only the *TAPBP* gene and demonstrated evasion of TAPBP-KO hiPSC-CMs from CTLs without inducing the innate immune response by NK cells. TAPBP-KO hiPSC-CMs represent a new cell source for cell-based therapies as a universal donor cell with simple gene editing.

### Limitations of the study

Hypoimmunogenic hiPSC-CMs were established by disrupting TAPBP, which reduced their HLA class I expression and low immunogenicity potential was demonstrated. However, the present study was limited to evaluating immune responses under IFN-γ-induced inflammatory conditions. In patients with advanced heart failure undergoing hiPSC-CMs transplantation, the myocardial microenvironment is characterized by multiple inflammatory cytokines and immune mediators. Therefore, our *in vitro* model does not fully recapitulate the complexity of the clinical inflammatory conditions, and future studies using more physiologically relevant inflammatory conditions will be necessary. Also, the *in vivo* therapeutic effect of TAPBP-KO hiPSC-CMs was not evaluated due to limitations in evaluating the immunological reaction caused by species-mediated immunological rejection.^42^ Several *in vivo* studies have been recently reported on evaluating the therapeutic effects of hiPSC-CMs in rodents, pigs, and NHPs, resulting in positive outcomes under immunosuppression.^9^^,^^43^^,^^44^^,^^45^ Clinical trials with hiPSC-CMs in patients with severe heart failure are being conducted.^10^ To evaluate the benefits of hypoimmunogenic cells, humanized mouse models are considered the most promising platform. However, current models have several limitations. Species-specific differences in cytokine signaling and immune development result in the incomplete reconstitution of human immune functions.^46^^,^^47^ In addition, impaired lymphoid organogenesis and the persistence of the murine innate immune system may compromise the evaluation of human adaptive and innate immune responses. Xenotransplantation using NHPs represents another potential approach; however, direct comparisons may be hindered by differences in differentiation protocols between human and NHP-derived iPSC-CMs.^8^^,^^48^ If advanced humanized models that overcome current limitations of xenotransplantation systems without requiring immunosuppressants were developed, they would provide an ideal platform for *in vivo* evaluation, enabling the assessment of long-term engraftment, immune evasion, and functional benefits of TAPBP-KO hiPSC-CMs.

## Resource availability

### Lead contact

Requests for further information, resources, and reagents should be directed to and fulfilled by the lead contact, Shugo Tohyama (shugo.tohyama@fujita-hu.ac.jp).

### Materials availability

This study did not generate new unique reagents.

### Data and code availability

• Data: Data reported in this study will be shared by the lead contact upon request.
• Code: This paper does not report original code.
• Other items: Any additional information required to reanalyze the data reported in this paper is available from the lead contact upon request.

## Acknowledgments

We thank Tomoko Haruna, Yumeko Konno, Yoko Shimizu, Sayaka Kanaami, and Noriko Kabasawa for their technical assistance with cell preparation and culture (Heartseed Inc. and Fujita Health University). We also thank Nanoko Sakamoto, Taijun Moriwaki, Kotaro Haga, Tomohiko C. Umei, Yuika Morita-Umei, and Masashi Nakamura, and other members for their advice and technical assistance (Fujita Health University). hiPSCs used in this study were distributed from the Center for hiPSCs research and application, 10.13039/501100005683Kyoto University, Japan under the collaboration. This work was mainly supported by Japan 10.13039/501100001691JSPS KAKENHI, Japan (grant 23K27683 and 25K21785 to S.T.) and a research grant from Heartseed Inc., and partly supported by 10.13039/100018456KISTEC, Japan (to S.T.).

## Author contributions

S.T., T.N., and K.F. conceptualized and designed the study; R.O. established TAPBP-KO hiPSCs; K.M., Y.K., and Y.Y. manufactured hiPSC-CMs; Y.Y., R.O., K.M., S.F. Y.K., Y.S., and H.T. conducted analysis of hiPSC-CMs characteristics; K.S., Y.K., T.Y., M.S., T. S., N.T., and K.O. conducted analysis of the immunological responses from CTLs and NK cells; Y.Y. and T.S. wrote the manuscript; and T.N. and S.T. supervised the study.

## Declaration of interests

Y.Y., R.O., K.S., and K.M. are the employees of Heartseed Inc. K.F. is the CEO of Heartseed Inc. S.T. is an advisor of Heartseed Inc. S.T. and K.F. own equity in Heartseed Inc.

## STAR★Methods

### Key resources table

**Table:** 

REAGENT or RESOURCE	SOURCE	IDENTIFIER
**Antibodies**		

HLA class I (FITC anti-human HLA-A, B, C Antibody)	BioLegend	# 311403 RRID:AB_314872
HLA class II (FITC anti-human HLA-DR, DP, DQ Antibody)	BioLegend	# 361705 RRID:AB_2563191
FITC Mouse IgG2a, κ Isotype Ctrl (FC) Antibody	BioLegend	# 400210 RRID:AB_326458
SSEA-4 Antibody, anti-human, PE	Miltenyi Biotec	# 130-122-958 RRID:AB_2811416
TRA-1-60 Antibody, anti-human, PE	Miltenyi Biotec	# 130-122-965 RRID:AB_2801990
REA Control Antibody, human IgG1, PE	Miltenyi Biotec	# 130-113-450 RRID:AB_2733892
Tapasin (E6P2Z) Rabbit Monoclonal Antibody	Cell signaling technology	# 66382T
GAPDH Monoclonal Antibody (6C5)	Thermo Fisher Scientific	# AM4300 RRID:AB_2536381
Anti-Rabbit Detection Module	ProteinSimple	# DM-001 RRID:AB_3095297
Anti-Mouse Detection Module	ProteinSimple	# DM-002 RRID:AB_3095298
Cardiac Troponin T Antibody, anti-human/mouse/rat, FITC	Miltenyi Biotec	# 130-119-575 RRID:AB_2751735
REA Control Antibody, human IgG1	Miltenyi Biotec	# 130-113-449 RRID:AB_2733688
Zombie NIR™ Fixable Viability Kit	BioLegend	# 423105
Oct3/4 Antibody (C-10)	Santa Cruz Biotechnology	# sc-5279 RRID:AB_628051
Anti-Nanog antibody	Abcam	# ab21624 RRID:AB_446437
Anti-Cardiac Troponin T antibody [1C11]	Abcam	# ab8295 RRID:AB_306445
Anti-Sarcomeric Alpha Actinin antibody	Abcam	# ab137346 RRID:AB_2909405
Anti-Myosin Light Chain 2 antibody	Abcam	# ab48003 RRID:AB_2282315
Donkey anti-Mouse IgG (H+L) Highly Cross-Adsorbed Secondary Antibody, Alexa Fluor™ 488	Thermo Fisher Scientific	# A-21202 RRID:AB_141607
Donkey anti-Mouse IgG (H+L) Highly Cross-Adsorbed Secondary Antibody, Alexa Fluor™ 546	Thermo Fisher Scientific	# A10036 RRID:AB_11180613
Goat anti-Rabbit IgG (H+L) Cross-Adsorbed Secondary Antibody, Alexa Fluor™ 488	Thermo Fisher Scientific	# A-11008 RRID:AB_143165
Goat anti-Rabbit IgG (H+L) Highly Cross-Adsorbed Secondary Antibody, Alexa Fluor™ 546	Thermo Fisher Scientific	# A-11035 RRID:AB_2534093
Donkey anti-Mouse IgG (H+L) Highly Cross-Adsorbed Secondary Antibody, Alexa Fluor™ 647	Thermo Fisher Scientific	# A-31571 RRID:AB_162542
MitoTracker™ Red FM	Thermo Fisher Scientific	# M22425
Human HLA-C MAb (Clone DT-9)	R&D Systems	# MAB10394-SP RRID:AB_3657237
HLA-F Monoclonal Antibody	Proteintech	# 66819-1-IG RRID:AB_3672923
PE anti-human HLA-E Antibody	BioLegend	# 342603 RRID:AB_1659250
PD-L1 (PE anti-human CD274 (B7-H1, PD-L1) Antibody)	BioLegend	# 393607 RRID:AB_2749924
PD-L2 (APC anti-human CD273 (B7-DC, PD-L2) Antibody)	BioLegend	# 345507 RRID:AB_2162177
PE Mouse IgG1, κ Isotype Ctrl (FC) Antibody	BioLegend	# 400114
APC Mouse IgG1, κ Isotype Ctrl (FC) Antibody	BioLegend	# 400122
Human ULBP-1 Phycoerythrin MAb	R&D Systems	FAB1380P RRID:AB_2687471
Mouse IgG2A Phycoerythrin Isotype Control	R&D Systems	IC003P RRID:AB_357245
MICA/MICB Monoclonal Antibody for FC	Proteintech	# APC-65161 RRID:AB_2883034
IgG2a Isotype Control Monoclonal Antibody for FC	Proteintech	# APC-65208 RRID:AB_2883049
APC anti-human CD8 Antibody	BioLegend	# 344722 RRID:AB_2075388
APC anti-human CD107a (LAMP-1) Antibody	BioLegend	# 328619 RRID:AB_1279057
PE anti-human CD56 (NCAM) Antibody	BioLegend	# 318306 RRID:AB_604101
BD Pharmingen™ APC Mouse Anti-Human CD16	BD Pharmingen	# 561248 RRID:AB_10612010
BD Pharmingen™ FITC Mouse Anti-Human CD16	BD Pharmingen	# 560996 RRID:AB_10562386
Brilliant Violet 421™ anti-human CD8 Antibody	BioLegend	# 344748 RRID:AB_2629584
PE anti-human CD3 Antibody	BioLegend	# 300456 RRID:AB_2564150

**Biological samples**		

PBMC	C.T.L.	# CTL-CP1 # CTL-UP1

**Chemicals, peptides, and recombinant proteins**		

iMatrix511	Nippi	# 892-012
iMatrix211	Nippi	# 892-062
Modified StemFit medium AS103C	Ajinomoto Co.	N/A
Modified StemFit medium AS301	Ajinomoto Co.	N/A
Modified StemFit medium AS501	Ajinomoto Co.	N/A
TrypLE Select	Thermo Fisher Scientific	#12563-011
T4 DNA ligase	NIPPON GENE	# 311-00404
Y-27632	FUJIFILM Wako Pure Chemical	# 034-24024
Cell Staining Buffer	BioLegend	# 420201
RIPA buffer	NACALAI TESQUE	# 08714
CHIR99021	FUJIFILM Wako Pure Chemical	# 034-23103
BMP4	R&D Systems	# 314-BP
IWR-1	Sigma-Aldrich	# 10161
Orlistat	Sigma-Aldrich	# O4139
2.5 g/L Trypsin EDTA	NACALAI TESQUE	# 35554-64
CryoStor CS10	STEMCELL Technologies	# 07930
4% Paraformaldehyde	Muto Pure Chemicals	# 33111
Triton X-100 (Polyoxyethylene Octylphenyl Ether)	FUJIFILM Wako Pure Chemical	# 168-11805
D-PBS (-)	FUJIFILM Wako Pure Chemical	# 049-29793
HBSS, calcium, magnesium, no phenol red	Thermo Fisher Scientific	# 14025092
Sodium pyruvate solution	Sigma-Aldrich	# S8636
MEM-α	Thermo Fisher Scientific	# 12571-048
FBS	Sigma-Aldrich	# 172012
Human Fibronectin	Sigma-Aldrich	# F0895
Cal520	AAT Bioquest	# 21131
pluronic F-127	AAT Bioquest	# 20053
ReadiUseTM probenecid	AAT Bioquest	# 20062
Isoproterenol	Sigma-Aldrich	# I6504
human CD8 microbeads	Miltenyi Biotec	# 130-045-201
AIM V™ Medium	Thermo Fisher Scientific	# 12055091
Human Serum	Sigma-Aldrich	# H4522
Recombinant Human IL-2 Protein	NIPRO	# 87-890
Recombinant Human IL-15	Peprotech	# 200-15
Phytohemagglutinin	FUJIFILM Wako Pure Chemical	# 151884
NK MACS® Medium, human	Miltenyi Biotec	# 130-114-429
RPMI-1640	FUJIFILM Wako Pure Chemical	# 183-02023
Calcein-AM	FUJIFILM Wako Pure Chemical	# 349-07201

**Critical commercial assays**		

MycoAlert	Lonza	# LT07-218
ReliaPrep™ RNA Cell Miniprep System	Promega	# Z6012
Superscript™ First-Strand Synthesis System	Thermo Fisher Scientific	# 18090050
CellTrace™ CFSE Cell Proliferation Kit, for flow cytometry	Thermo Fisher Scientific	# C34554
NK Cell Isolation Kit, human	Miltenyi Biotec	# 130-092-657
BD™ ELISPOT Human IFN-γ ELISPOT Set	BD Biosciences	# 551849

**Experimental models: Cell lines**		

FfI14	CiRA	N/A
K562	RIKEN BRC Cell Bank	RCB1897

**Oligonucleotides**		

gRNA-TAPBP-KO F : caccgaaccaacactcgatcaccg R : aaaccggtgatcgagtgttggttc	FASMAC	N/A
GAPDH	Thermo Fisher Scientific	Hs02758991
MYL2	Thermo Fisher Scientific	Hs00166405
MYL7	Thermo Fisher Scientific	Hs01085598
TNNI3	Thermo Fisher Scientific	Hs00165957
TNNI1	Thermo Fisher Scientific	Hs00913333
MYH7	Thermo Fisher Scientific	Hs01110632
MYH6	Thermo Fisher Scientific	Hs01101425

**Recombinant DNA**		

pX330-U6-Chimeric_BB-CBh-hSpCas9 (pX330)	Cong et al.^49^	Addgene # 42230 RRID:Addgene_42230

**Software and algorithms**		

Kaluza	Beckman Coulter	https://www.beckman.jp/flow-cytometry/software/kaluza/downloads
CytoFLEX	Beckman Coulter	https://www.beckman.jp/flow-cytometry/research-flow-cytometers/cytoflex
FACS Aria II	BD Biosciences	https://www.bdbiosciences.com/ja-jp/products/instruments/flow-cytometers/research-cell-sorters/bd-facsaria-iii
FACS Canto II	BD Biosciences	https://www.bdbiosciences.com/ja-jp/products/instruments/flow-cytometers/clinical-cell-analyzers/facscanto
DIVA software	BD Biosciences	https://www.bdbiosciences.com/ja-jp/products/software/instrument-software/bd-facsdiva-software
APX100	Evident	https://evidentscientific.com/ja/products/inverted/apexview
TruSight	Evident	https://evidentscientific.com/ja/landing/trusight
BZ-X710	Keyence	https://www.keyence.co.jp/products/microscope/fluorescence-microscope/bz-x700/models/bz-x710/
QuantStudio™ 12 Flex Real-Time PCR System	Applied Biosystems	https://www.thermofisher.com/jp/ja/home/life-science/pcr/real-time-pcr/real-time-pcr-instruments/quantstudio-systems/models/quantstudio-12-flex.html
FDSS/μCELL	Hamamatsu Photonics K.K.	https://www.hamamatsu.com/jp/ja/product/life-science-and-medical-systems/kinetic-plate-imager/C13299-02.html
JESS simple western system	ProteinSimple	https://proteinsimple.jp/simple_western_overview.html
GraphPad Prism 11	GraphPad Software	https://www.graphpad.com/updates/prism-11-0-0-release-notes

### Experimental model and study participant details

#### Ethical approval

The use of human PBMCs was approved by the Ethics Committee of the National Cancer Center (reference 2015-273), and the use of HLA-homozygous hiPSCs was approved by the Ethics Committee of Fujita Health University (reference HM25-670).

#### Cultivation of hiPSCs

Human leukocyte antigen (HLA) haplotype-homozygous hiPSC lines (FfI14) were provided by the Center for iPSCs Research and Application, Kyoto University.^14^^,^^31^ hiPSCs were maintained and expanded on iMatrix511 (Nippi)-coated culture plates using a modified StemFit medium AS103C, hiPSC expansion (Ajinomoto Co.) based on the previous report.^50^ Cells were detached with TrypLE Select (Thermo Fisher Scientific) and passaged every 3–4 d. Cell numbers were counted using trypan-blue staining. Mycoplasma contamination was confirmed every 4 m using MycoAlert (Lonza).

#### Generation of TAPBP-KO hiPSCs

The plasmid, pX330-U6-Chimeric_BB-CBh-hSpCas9 (pX330), which contains gRNA and the *Cas9* gene under an RNA POL III promoter and a constitutive RNA Pol II promoter, respectively, was obtained from Addgene.^49^ The gRNA sequence was purchased from FASMAC. The gRNA sequence was inserted in the pX330 by the T4 DNA ligase (NIPPON GENE) (pX330-TAPBP). hiPSCs were pretreated with a ROCK inhibitor (Y-27632; FUJIFILM Wako Pure Chemical) at 10 μM for at least 1 h before electroporation. hiPSCs were dissociated into single cells and transduced with pX330-*TAPBP* using a NEPA 21 electroporator (poring pulse: pulse voltage, 125 V; pulse width, 5 ms; pulse number, 2; Nepagene). A total of 56 clones were isolated for expansion, and the lowest HLA class I-expressed clone was selected. The HLA was stained with HLA class I and HLA class II (BioLegend) with or without IFN-γ stimulation for 48 h, cells were stained for stemness markers with SSEA-4 and TRA-1-60 (Miltenyi Biotec). The population was measured using CytoFLEX (Beckman Coulter) and analyzed by Kaluza (Beckman Coulter).

#### Western blot

The cell lysate was prepared with RIPA buffer (NACALAI TESQUE) and denatured at 95°C for 5 m. The immunodetection was performed automatically using the JESS simple western system (ProteinSimple) staining by the following primary antibodies: anti-tapasin antibody (Cell Signaling Technology) and GAPDH (Thermo Fisher Scientific); and the following secondary antibodies: Anti-Rabbit Detection Module and Anti-Mouse Detection Module (ProteinSimple).

#### Cardiomyocyte differentiation

hiPSCs were differentiated into cardiomyocytes as described in previous reports.^27^^,^^28^^,^^51^ Briefly, the differentiation was initiated by the supplementation of CHIR99021 (FUJIFILM Wako Pure Chemical) and BMP4 (R&D Systems), then the cells were supplemented with IWR-1 (Sigma). The cells were re-plated under supplementation with the fatty acid synthesis inhibitor, orlistat (Sigma), for 3 d and then CMs were metabolically selected in glucose- and glutamine-free medium supplemented with lactate, StemFit medium AS501 (Ajinomoto Co.).^26^^,^^27^^,^^29^^,^^52^ hiPSC-CMs were detached with Trypsin-EDTA (NACALAI TESQUE) and cryopreserved in Cryostor CS10 (STEMCELL Technologies). hiPSC-CMs were cultured for 1 w after thawing to allow recovery before use, thereby ensuring consistent cell quality and accurate cell counting.

The purity of hiPSC-CMs was measured by staining cardiac troponin T (Miltenyi Biotec) (1:50 dilution), followed by zombie-NIR staining (BioLegend) (1:100 dilution), fixation with 4% paraformaldehyde (4% PFA) and permeabilization with 0.1% TritonX-100 in phosphate-buffered saline (PBS). The population was measured using CytoFLEX and analyzed by Kaluza.

#### Immunocytochemistry staining

The cells were fixed with 4% PFA and permeabilized with 0.1% TritonX-100 in PBS. Primary antibody (1:100 dilution) was added at 4 °C overnight, followed by staining with the second antibody (1:100 dilution) at 4 °C for another night. Hoechst (1:200) was stained for 15 m at approximately 25 °C in the dark. Cells were photographed using a BZ-X710 microscope (Keyence), or APX100 and deconvolution with TruSight (Evident). Immunofluorescence staining of cells was performed using the following primary antibodies: anti-OCT-3/4 (Santa Cruz Biotechnology), anti-NANOG (Abcam), anti-cardiac Troponin T (Abcam), anti-actinin (Abcam) and anti-MLC2v (Abcam); and the following secondary antibodies: anti-rabbit or anti-mouse IgG conjugated with Alexa Flour™ 488 or 546 (all from Thermo Fisher Scientific). The mitochondria staining was carried out with MitoTracker™ Dyes (Thermo Fisher Scientific) in Hanks’ balanced salt solution (Thermo Fisher Scientific) at 37 °C for 30 m prior to the 4% PFA fixation.

#### RNA extraction and quantitative RT-PCR analysis

Total RNA samples were isolated using a ReliaPrep™ RNA Cell Miniprep System (Promega), according to the manufacturer’s instructions. cDNA was synthesized using the Superscript™ First-Strand Synthesis System (Thermo Fisher Scientific). Quantitative RT-PCR was performed using the QuantStudio™ 12 Flex Real-Time PCR System (Applied Biosystems), with *MYL2* (Hs00166405), *MYL7* (Hs01085598), *TNNI3* (Hs00165957), *TNNI1* (Hs00913333), *MYH7* (Hs01110632), *MYH6* (Hs01101425) TaqMan® probes (Thermo Fisher Scientific). The mRNA levels were normalized to those of *GAPDH* (Hs02758991).

#### Flow cytometry analysis for immunogenicity-associated molecules

The immunogenicity-associated molecules on hiPSC-CMs were evaluated by flow cytometry with or without IFN-γ stimulation for 48 h. For HLA-C and HLA-F detection, cells were stained with primary antibodies diluted 1:100 in cell signaling buffer at 4 °C for 20 m, followed by incubation with an Alexa Fluor 647-conjugated anti-mouse IgG secondary antibody. Similarly, HLA-E, PD-L1, PD-L2, ULBP, and MICA/MICB were stained at a 1:100 dilution under the same conditions. Cell populations were analyzed using a CytoFLEX flow cytometer and the data were processed with Kaluza software.

#### Contractile analysis

hiPSC-CMs were cultured for approximately 1 w in a culture medium containing 2.5% fetal bovine serum (FBS) and 2 mM sodium pyruvate in MEMα on a Fibronectin (Sigma-Aldrich)-coated plate. On the day of the assay, the cells were incubated in 5 mM Cal520 (AAT Bioquest) in Hanks’ balanced salt solution supplemented with pluronic F-127 (AAT Bioquest) and probenecid (AAT Bioquest) at 37 °C for 1 h. The expression of calcium transient was measured using FDSS/μCELL (Hamamatsu Photonics K.K.). Isoproterenol (MERCK) was added to the culture medium.

The contractile characteristics were also analyzed utilizing SI8000 live-cell motion imaging system (Sony Corporation) as described in the previous report.^53^^,^^54^

#### Establishment of allo-reactive cytotoxic T lymphocytes (CTLs)

To establish CTL clones with reactivity against allogeneic FfI14, CD8^+^ cells were positively purified from PBMC with HLA haplotypes different from FfI14 (HLA-A 24:02 and HLA-B 52:01) using magnetic-activated cell sorting (MACS) with human CD8 microbeads (Miltenyi Biotec). Purified CD8^+^ cells were labeled with 5 μM carboxyfluorescein succinimidyl ester (CFSE) using the CellTrace CFSE Cell Proliferation Kit (Thermo Fisher Scientific) and then co-cultivated with irradiated (100Gy) allogeneic PBMC with the same HLA haplotypes as FfI14 in AIM-V (Thermo Fisher Scientific) supplemented with 5% AB serum (Sigma-Aldrich).

Two days after co-cultivation, Recombinant IL-2 (NIPRO) and IL15 (Peprotech) were added at final concentration of 50 U/mL and 10 ng/mL, respectively. Seven days after mixed culture, cells were harvested, stained with APC-conjugated anti-CD8 monoclonal antibody (mAb, BioLegend). The CFSE-negative CD8-positive cells were sorted using FACS Aria II (BD Biosciences) as a proliferating allo-reactive CTL line. Purified allo-reactive CTL lines were re-expanded with irradiated (100 Gy) feeder PBMC in the presence of 50 U/mL IL-2, 10 ng/mL IL15, and 1 μg/mL PHA (FUJIFILM Wako Pure Chemical).

After expansion, allo-reactive CTL lines were co-cultivated with FfI14 cells, and a degranulation assay was conducted by staining with anti-CD107a mAb (BioLegend). After 4 h co-culture, CD107a- and CD8-positive T cells were isolated as single cells by sorting with FACS Aria II, expanded with feeder PBMC in the presence of cytokines and PHA, and used as FfI14-reactive CTL clones in subsequent experiments.

#### Isolation and expansion of NK cells

Using CD8^+^ microbeads and an NK cell Isolation kit (Miltenyi Biotec), NK cells were isolated from PBMC with HLA haplotype matched or mismatched with FfI14 cells, following the instructions in the manuals of each kit. The negatively purified NK fraction was expanded using an NK Cell Activation/Expansion kit (Miltenyi Biotec) in NK MACS medium (Miltenyi Biotec) supplemented with 5% human AB serum and 500 U/mL IL-2. The purity of the NK cell population was measured by the expression of CD56 (BioLegend) and CD16 (BD Pharmingen) using FACS Canto II (BD Biosciences) with DIVA software (BD Biosciences), and CD56^+^ NK cells were used for subsequent experiments.

#### CD107a degranulation assay

To assess the degranulation of effector cells associated with cellular cytotoxicity, 5 x 10^4^ target cells and 1 x 10^5^ effector cells with preparation medium (10% FBS RPMI-1640) were co-cultivated in a U-bottom 96-well plate in the presence of 4 μg/mL APC-conjugated anti-CD107a mAb for 3.5 h. After co-cultivation, cells were harvested, stained using BV421-conjugated anti-CD8 mAb and PE-conjugated anti-CD3 mAb (BioLegend). After fixation with 2.5% PFA, the CD107a-positive cells in the CD8^+^CD3^+^ gated population were measured using FACS Canto II with Diva software. In some experiments, to induce the expression of HLA, target cells were treated with 10 ng/mL IFN-γ 48 h before the assay.

#### ELISpot assay

The BD™ ELISpot Human IFN-γ ELISpot Set (BD Bioscience) was used according to the manufacturer's instructions. Briefly, the ELISpot plates were coated overnight with the anti-hIFN-γ mAb and subsequently blocked with preparation medium for 2 h. After blocking, 5 x 10^4^ target cells and 1 x 10^5^ effector cells with preparation medium were co-cultivated in ELISpot plates for 4 h. The IFN-γ production was detected as spots using biotin-conjugated antibody and StAv HRP. Using an ELIPhoto counter (MINERVATECH), the photograph of each well was captured, and the number of spots per well was measured. In some experiments, target cells were treated with 10 ng/mL IFN-γ 48 h before assays to induce the expression of HLA. Wells with excessive or indistinct spots were assigned a provisional value of 500.

#### *In vitro* cell killing assay

The *in vitro* cell killing assay was conducted as described previously.^55^ In brief, target cells were labeled with calcein-AM (DOJIN), washed, and then co-cultivated with effector cells, CTLs or NK cells, for 18 h or 4 h, respectively, at various ratios of effector to target cells in 96-half well plates. In some experiments, target cells were treated with 10 ng/mL IFN-γ 48 h before assays to induce the expression of HLA. The decay of fluorescence associated with the cell death of stained target cells was measured using the Terascan VPC system (Minerva Tech). The cytotoxicity was calculated by comparing the fluorescence intensity before and after co-cultivation:

(Equation 1) Cytotoxicity(%)={[(averagefluorescenceofthesamplewells−averagefluorescenceofthemaximalreleasecontrolwells)/(averagefluorescenceoftheminimalreleasecontrolwells−averagefluorescenceofthemaximalreleasecontrolwells)]}×100

#### Contractile analysis upon immunological reaction

Non-cryopreserved hiPSC-CMs were harvested and seeded at 2.5 x 10^3^ cells / well in 96-V plates for 1 w to form cardiac spheroids. Effector cells (CTLs (Donor A) or NK cells (Donor F)) were then added to each well at an E/T ratio of 1 for 21 h or 3 h, respectively. The beating rate was measured by the SI8000 live-cell motion imaging system.

### Method details

An HLA haplotype-homozygous hiPSC line (FfI14) was provided by the Center for iPS Cell Research and Application (CiRA), Kyoto University.^14^^,^^31^ Human Peripheral blood mononuclear cells (PBMCs) were obtained from healthy donors, being either purchased from Cellular Technology Limited (C.T.L.) or collected from volunteers who provided written informed consent for the use of their specimens for research purposes.

### Quantification and statistical analysis

All statistical analyses were performed using GraphPad Prism 11 (GraphPad Software Inc). Values are presented as mean ± SEM. Differences between groups were examined for statistical significance using unpaired or paired t-tests. Statistical significance was set at *p* < 0.05: ∗ *p* < 0.05 and ∗∗ *p* < 0.01, ∗∗∗ *p* < 0.001, ∗∗∗∗ *p* < 0.0001, and ns *p* > 0.05.
