# Supplementary material for: Generation of hypoimmunogenic cardiomyocytes from human induced pluripotent stem cells with TAPBP knockout
Source: iScience. 2026 Oct 17;29(11):117933. doi: 10.1016/j.isci.2026.117933 (PMC13695367; doi:10.1016/j.isci.2026.117933)
Supplement: Supplementary file 1 [file mmc1.pdf]

iScience, Volume *29*

Supplemental information

Generation of hypoimmunogenic cardiomyocytes from human induced pluripotent stem cells with TAPBP knockout

Yuki Yamamoto, Rei Ohno, Kayoko Shoda, Toshihiro Suzuki, Kuniko Momoi, Shohei Fujii, Yuka Kenmochi, Toshiaki Yoshikawa, Manami Shimomura, Nobuo Tsukamoto, Kazunobu Ohnuki, Hidenori Tani, Yusuke Soma, Keiichi Fukuda, Tetsuya Nakatsura, and Shugo Tohyama

Figure S1

A

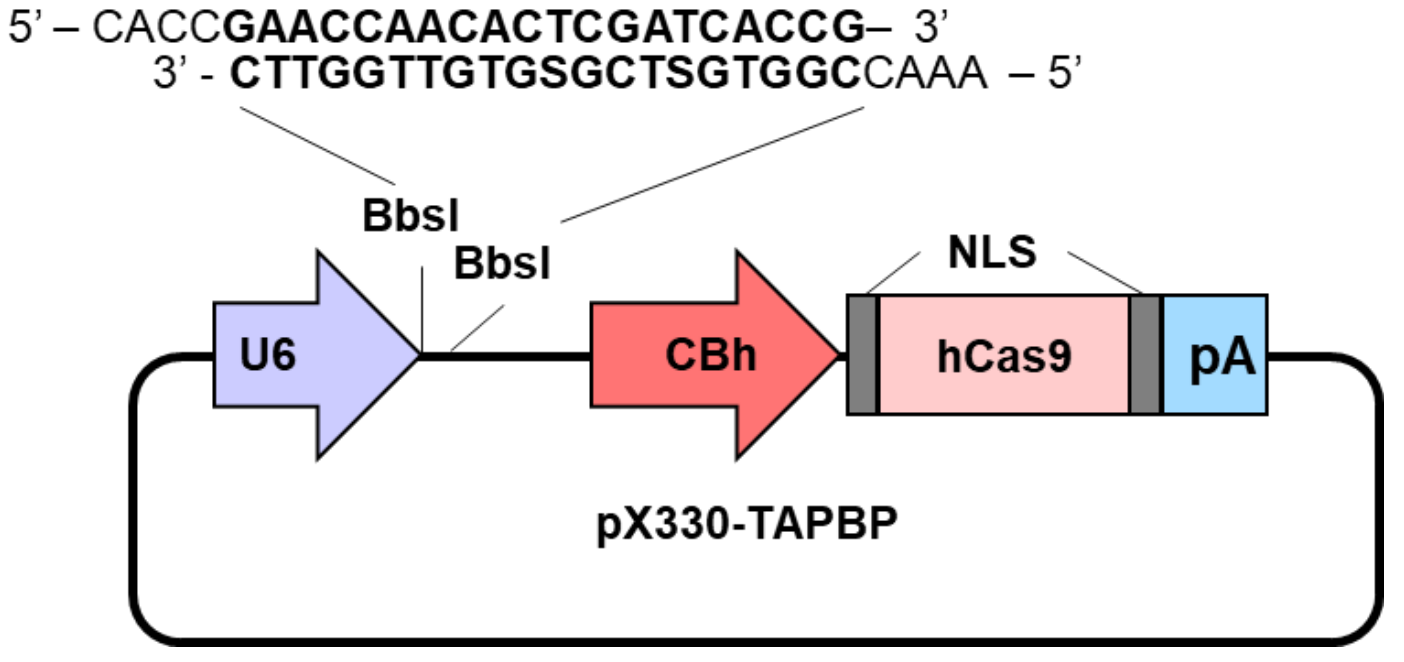

B

% HLA class I positive	# of clones
～20%	4
20-40%	7
40-60%	4
60-80%	17
80%～	24
total	56

C

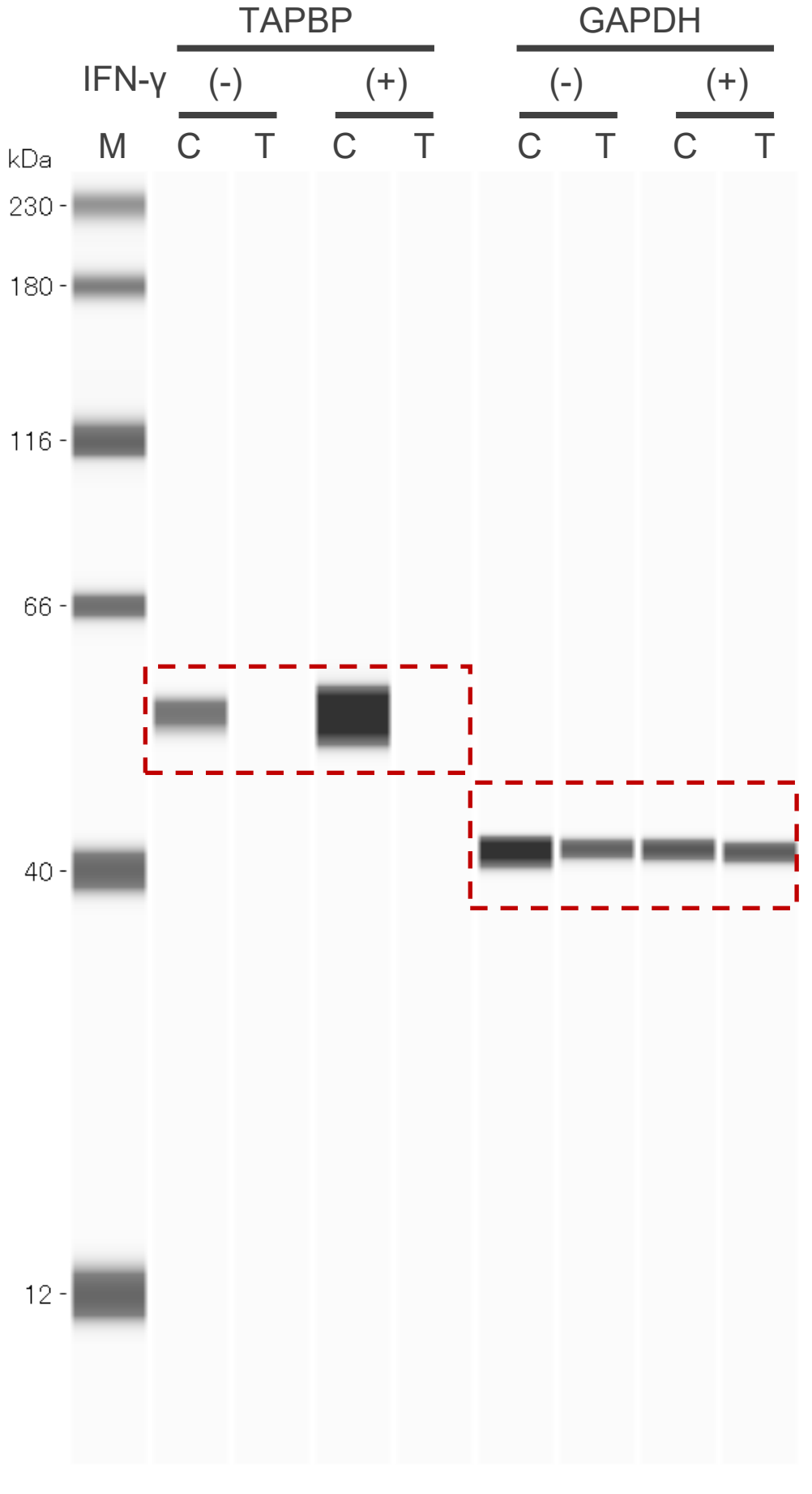

Figure S1. Details of generating TAPBP-KO hiPSCs, related to Figure 1.

(A) Schematic describing the plasmid construction of pX330-*TAPBP*

(B) The table shows the different expression of the HLA class I of TAPBP-KO hiPSCs. The threshold of the positivity was against isotype control

(C) Uncropped Western blot image. Lane designations: Molecular weight maker (M), control hiPSCs (C), and TAPBP-KO hiPSCs (T).

Figure S2

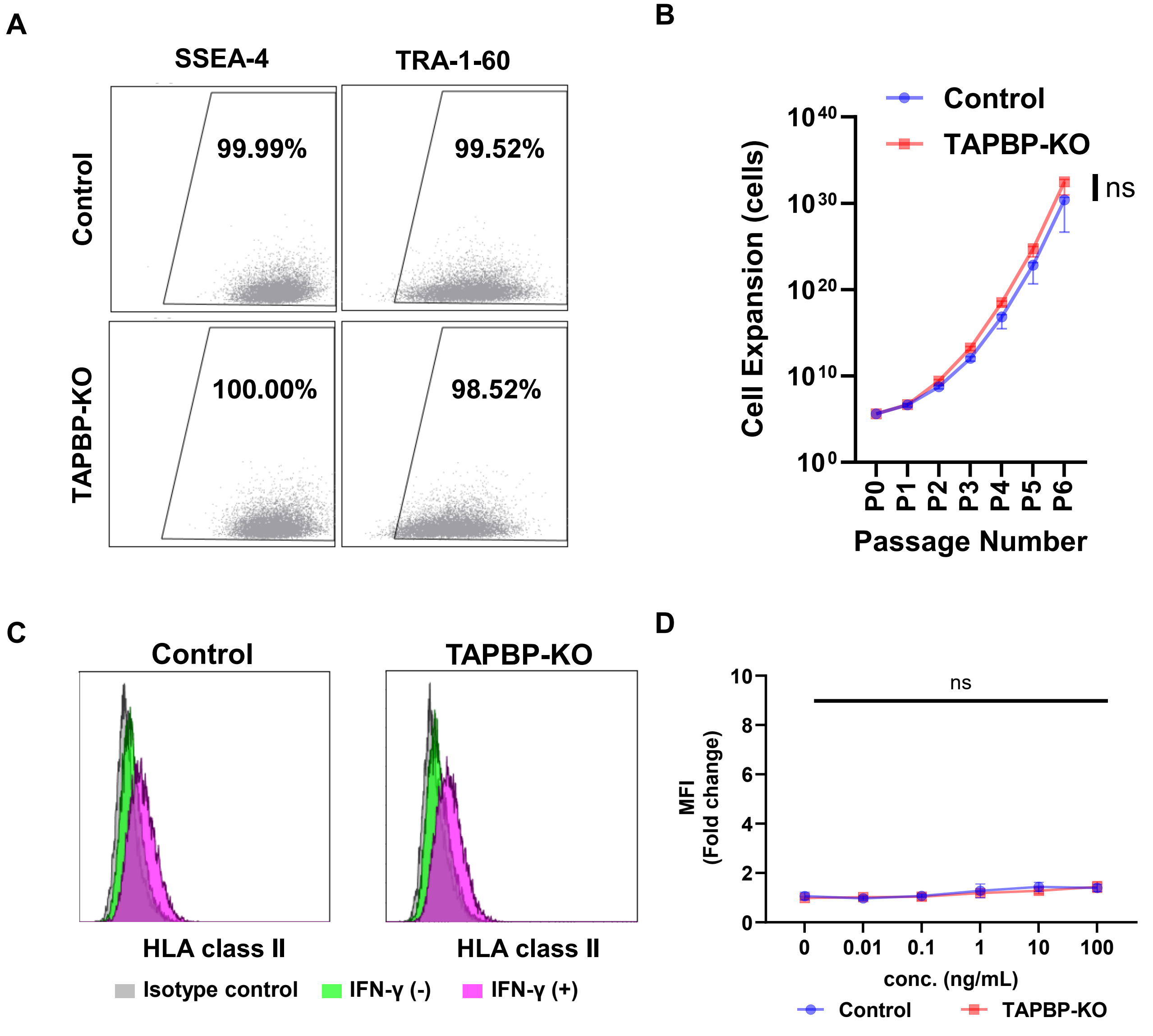

A
SSEA-4
TRA-1-60
Control
TAPBP-KO
99.99%
99.52%
100.00%
98.52%
B
Control
TAPBP-KO
ns
Cell Expansion (cells)
10^40
10^30
10^20
10^10
10^0
P0
P1
P2
P3
P4
P5
P6
Passage Number
C
Control
TAPBP-KO
HLA class II
HLA class II
Isotype control
IFN-γ (-)
IFN-γ (+)
D
ns
MFI
(Fold change)
10
8
6
4
2
0
0
0.01
0.1
1
10
100
conc. (ng/mL)
Control
TAPBP-KO

Figure S2. Characteristics of TAPBP-KO hiPSCs, related to Figure 1.

(A) Representative flow cytometry plots for the expression of stemness markers, SSEA-4 and TRA-1-60, in control and TAPBP-KO hiPSCs

(B) Cell expansion level was measured in control and TAPBP-KO hiPSCs. (n=3, independent experiments); ns, $p > 0.05$ using 2way ANOVA

(C) The representative histograms by flow cytometry show enhanced HLA class II expression by the treatment of IFN-γ (pink) compared with non-treatment (green) in both control and TAPBP-KO hiPSCs

(D) Dose-dependent response of HLA class II against IFN-γ stimulation was measured by flow cytometry (n=3, independent experiments). Each plot is shown by the relative mean fluorescence intensity (MFI) of HLA class II against isotype control; ns, $p > 0.05$ using an unpaired t-test

Figure S3

A

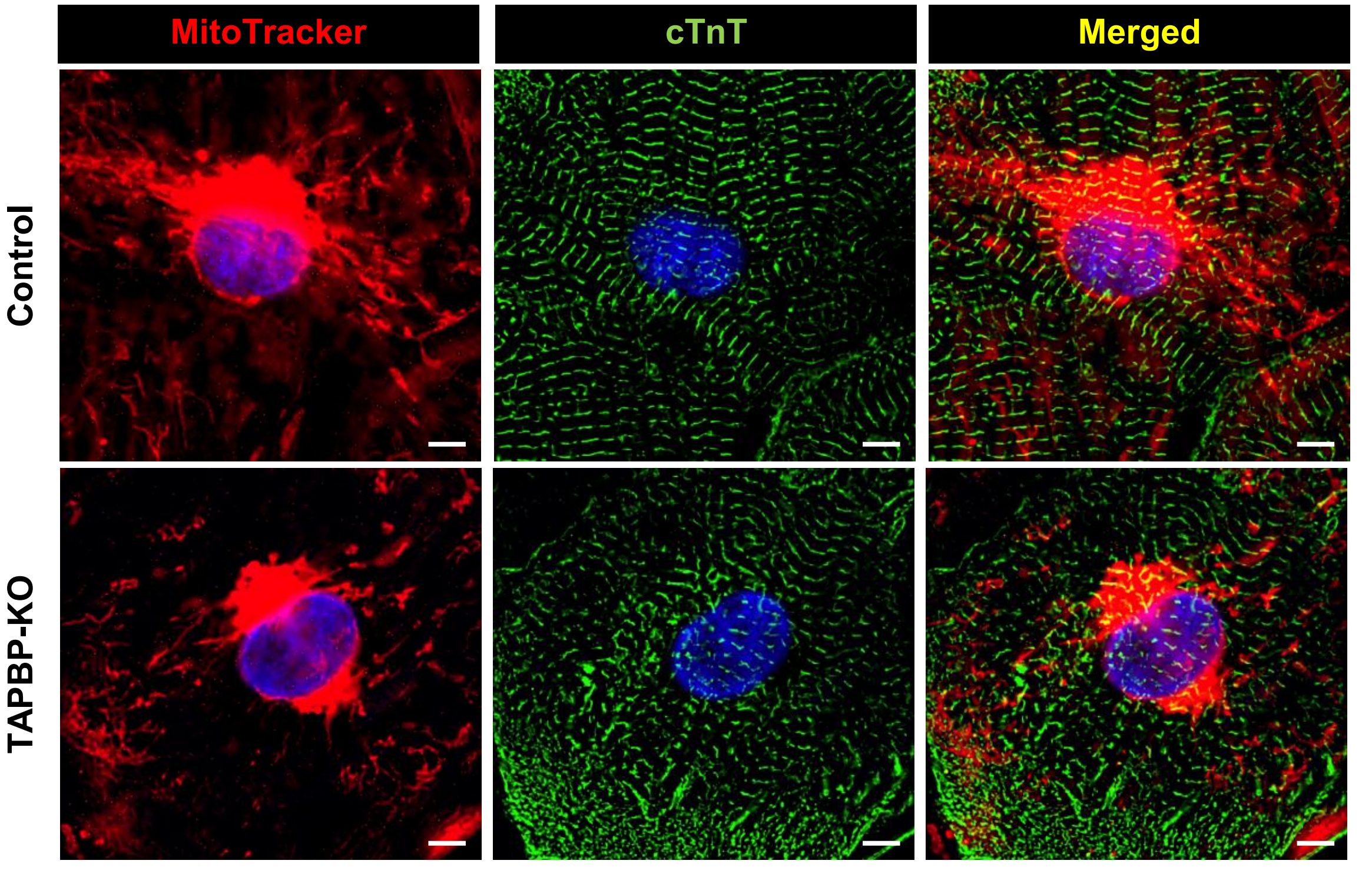
MitoTracker
cTnT
Merged
Control
TAPBP-KO

B

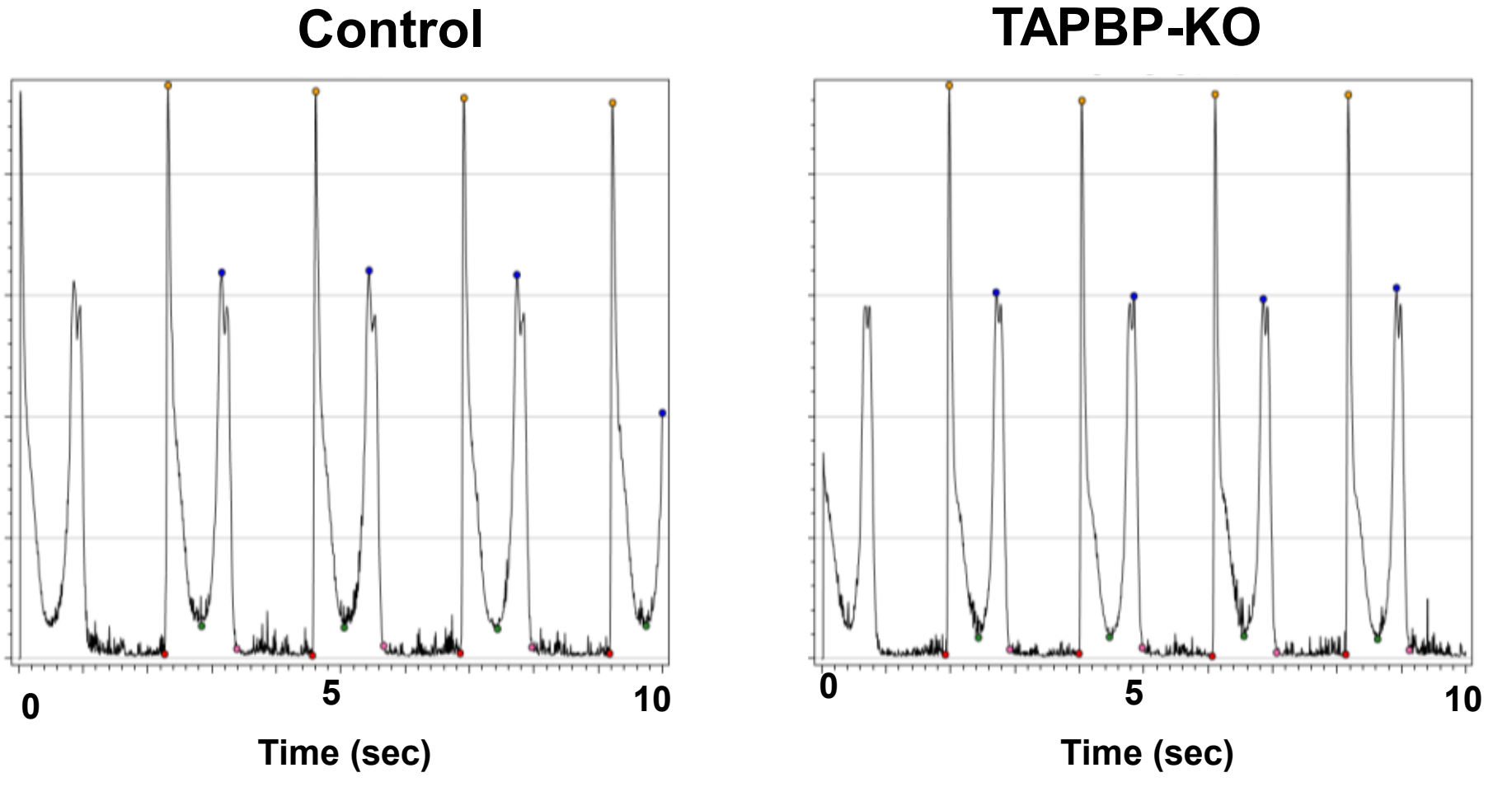
Control
TAPBP-KO
0
5
10
Time (sec)
0
5
10
Time (sec)

Figure S3. Characteristics of TAPBP-KO hiPSC-CMs, related to Figure 2.

(A) Representative immunostaining shows the mitochondria were gathered around the nuclei both in control and TAPBP-KO hiPSC-CMs. MitoTracker (red), cTnT (green), and Hoechst33432 (blue); scale bar = 5 μm

(B) Representative beating characteristics of hiPSC-CMs were recorded and analyzed by SI8000 live-motion imaging system

Figure S4

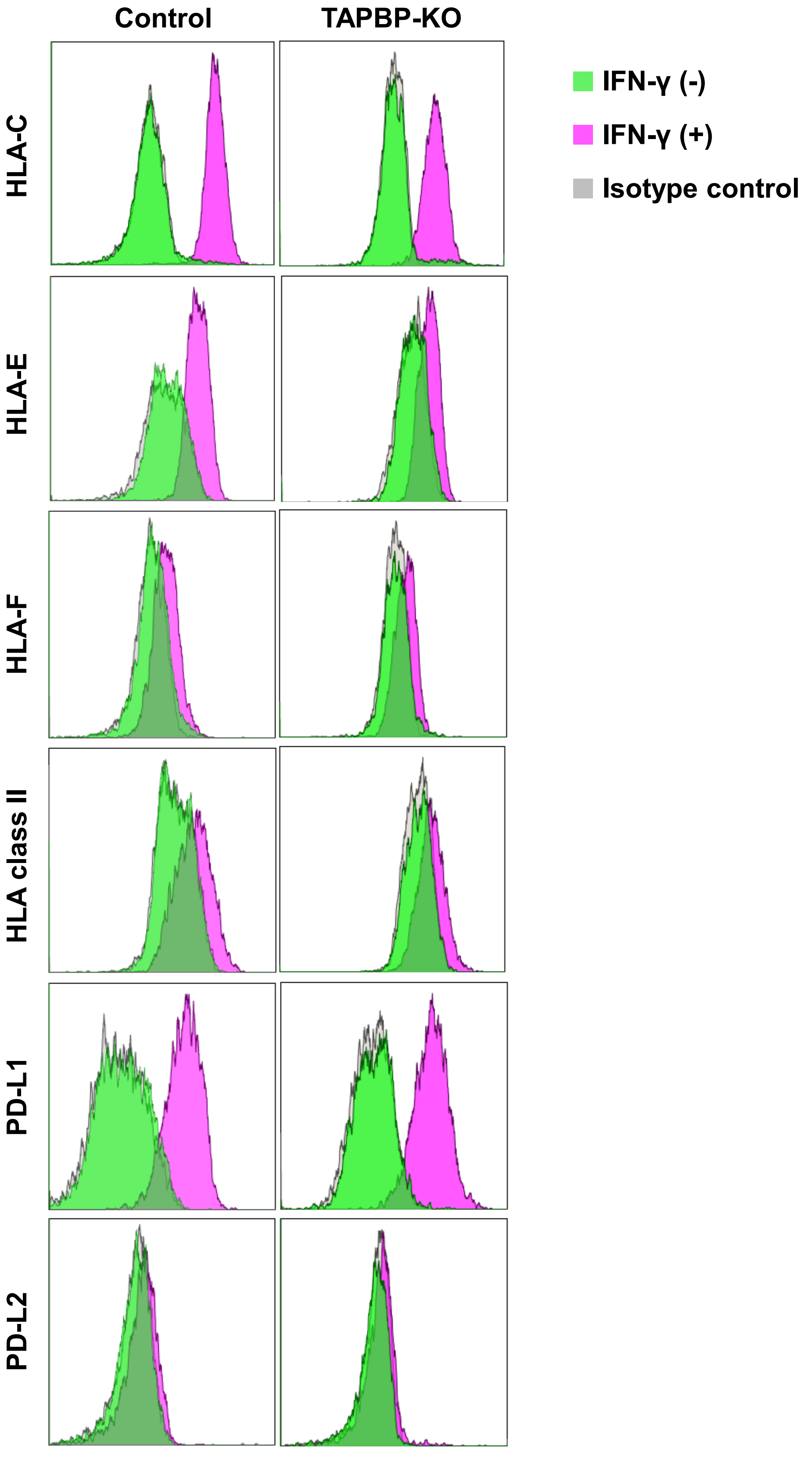

Control
TAPBP-KO
HLA-C
HLA-E
HLA-F
HLA class II
PD-L1
PD-L2
IFN-γ (-)
IFN-γ (+)
Isotype control

Figure S4. Expression of Immunogenicity-associated molecules, related to Figure 2.
Histograms represent the expression of immunogenicity-associated molecules in control and TAPBP-KO hiPSC-CMs with (pink) or without (green) IFN-γ stimulation.

Figure S5

A

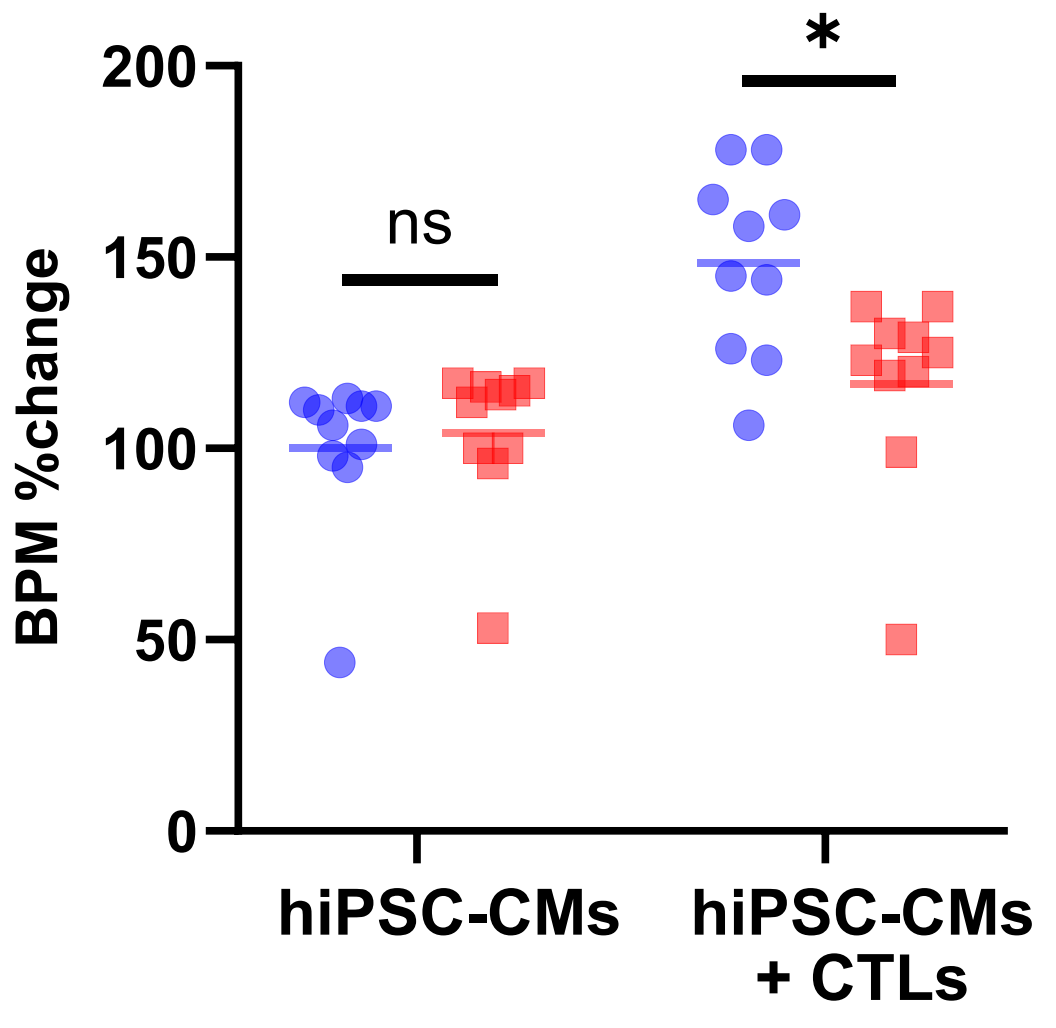

Figure S5. Change of beating upon immunological reaction from CTLs, related to Figure 3. Change in beating rate relative to control hiPSC-CMs upon co-culture with CTLs, normalized to control hiPSC-CMs without CTLs. The plots indicate data points for control (blue) and TAPBP-KO (red) hiPSC-CMs with or without CTLs, and the lines represent the mean; ns $p > 0.05$ and * $p < 0.05$ using unpaired t-tests (n=10).

Figure S6

A

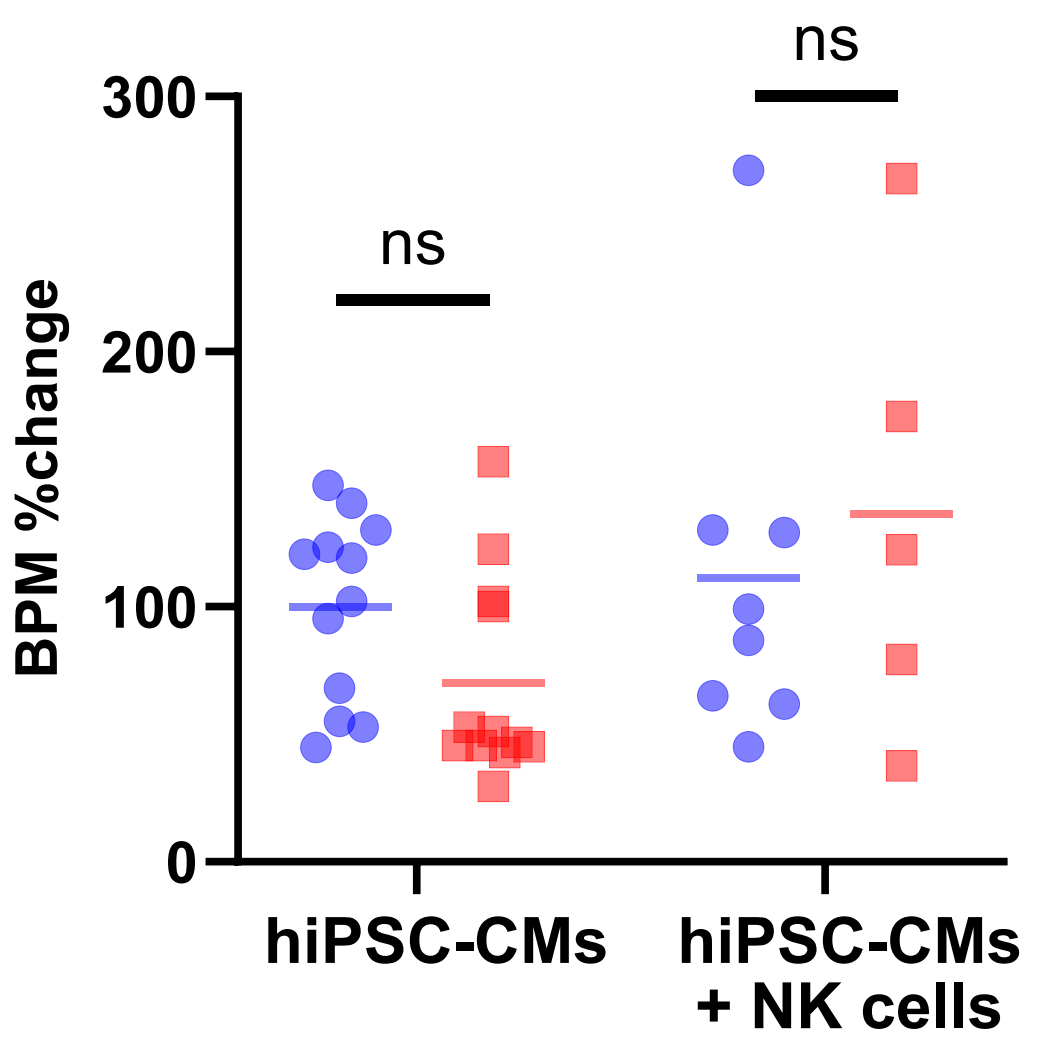
ns
ns
300
200
100
0
BPM %change
hiPSC-CMs
hiPSC-CMs
+ NK cells

B

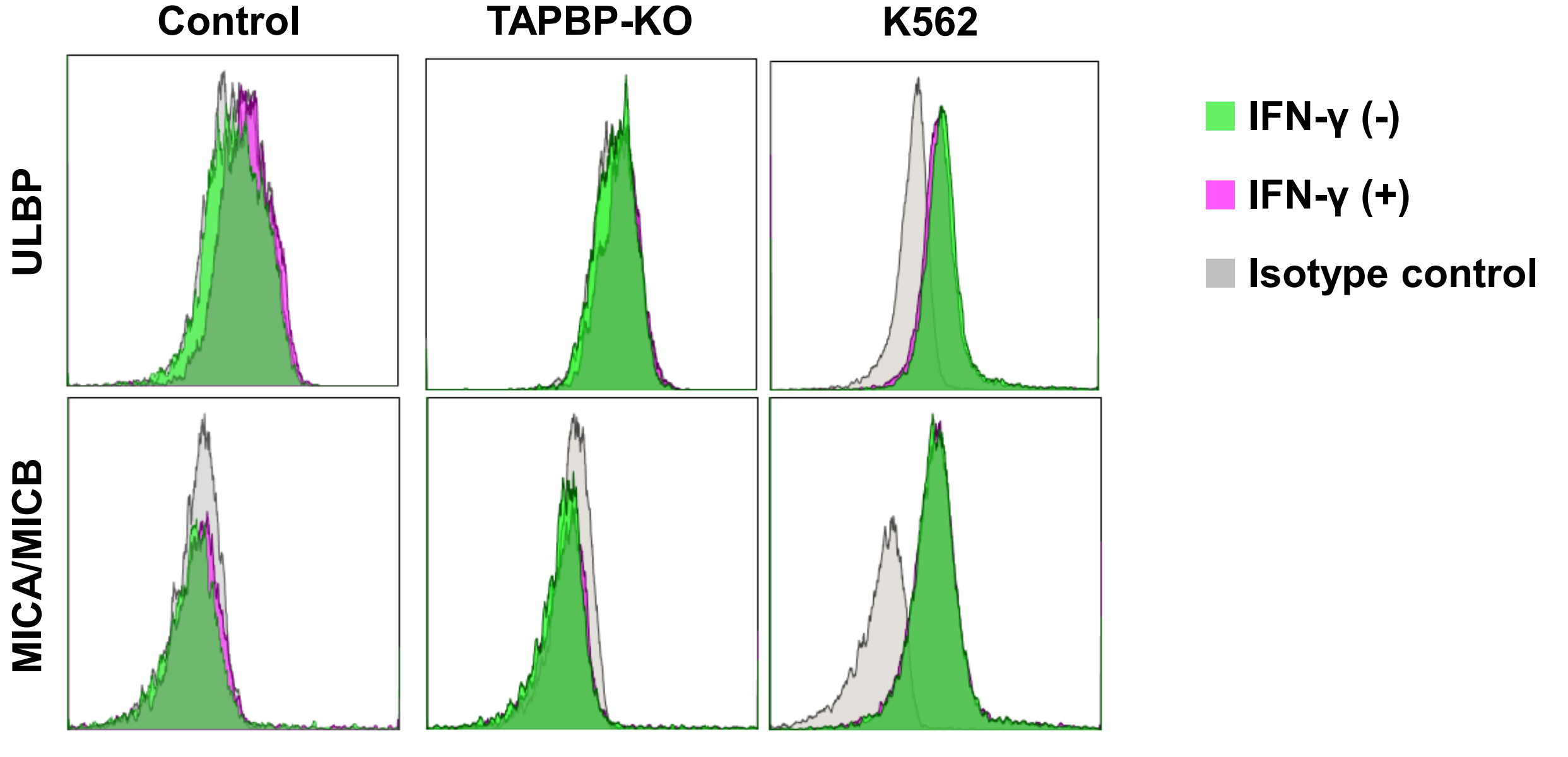
Control
TAPBP-KO
K562
ULBP
MICA/MICB
IFN-γ (-)
IFN-γ (+)
Isotype control

Figure S6. Change of beating upon immunological reaction from NK cells, related to Figure 4.

(A) Change of beating rate relative to control hiPSC-CMs upon co-culture with NK cells, normalized to control hiPSC-CMs without NK cells. The plots indicate data points for control (blue) and TAPBP-KO (red) hiPSC-CMs with or without NK cells, and the bars are presented as mean; ns $p > 0.05$ using unpaired t-tests. (hiPSC-CMs; n=12 for both control (blue) and TAPBP-KO (red) hiPSC-CMs, hiPSC-CMs + NK cells; n= 8 or 5 for control or TAPBP-KO hiPSC-CMs, respectively)

(B) The histograms represent the expression of NK activation ligands with (pink) or without (green) IFN-γ stimulation in both control and TAPBP-KO hiPSC-CMs, and K562.
